# Optimization of next-generation CXCR4 ligands for radiotheranostic applications

**DOI:** 10.3389/fphar.2026.1923347

**Published:** 2026-09-04

**Authors:** Itzel Reneé Astiazarán-Rascón, Joseph Lau, François Bénard

**Affiliations:** 1 Department of Basic and Translational Research, BC Cancer Research Institute, Vancouver, BC, Canada; 2 Department of Radiology, University of British Columbia, Vancouver, BC, Canada

**Keywords:** cancer, CXCR4, drug design, positron emission tomography, radiotheranostics

## Abstract

Radiotheranostic agents enable paired diagnostic imaging and targeted radiopharmaceutical therapy through the selection of appropriate radioisotopes. Radiotheranostics are typically composed of a targeting vector, a chelator or prosthetic group for radiolabeling and an optional linker for optimizing their pharmacokinetic properties. The chemokine receptor 4 (CXCR4) is a promising target for molecular imaging and therapy across a broad range of pathological conditions, including cancer, cardiovascular disorders, infectious and autoimmune diseases. The elucidation of CXCR4 crystal structures, together with extensive structure-activity relationship studies, has accelerated the design of novel small molecule and peptide-based CXCR4-targeted radiopharmaceuticals. These molecules have served as scaffolds that can be chemically modified to optimize their pharmacokinetic properties and maximize accumulation at the target organ. In oncology, the peptide-based theranostic pair Pentixafor/Pentixather has advanced into early-stage clinical evaluations for imaging and therapy. This review summarizes the current landscape of CXCR4 radiopharmaceuticals, and highlights key structural features and chemical modifications that lead to enhanced receptor affinity as well as improved pharmacokinetic and *in vivo* targeting properties for theranostic applications.

## 1 Introduction

Chemokines and their receptors regulate diverse physiological and pathological processes through a highly coordinated signaling network. More than 50 human chemokines and 20 chemokine receptors have been reported (Zlotnik and Yoshie, 2012). Chemokine receptors belong to the family of seven transmembrane G protein-coupled receptors (GPCRs), which bind to multiple chemokines to coordinate cell migration, immune surveillance, and homeostasis (Zlotnik and Yoshie, 2012; Griffith et al., 2014; Mollica Poeta et al., 2019). Among these, CXC-chemokine receptor 4 (CXCR4) is a well-characterized GPCR that binds selectively to its cognate ligand C-X-C motif chemokine ligand 12 (CXCL12), also known as stromal-derived factor-1α (SDF-1α). CXCR4 is expressed in human stem and progenitor cells, differentiated immune cells, epithelial cells and fibroblasts. CXCR4 activation triggers pleiotropic functions through G_i_ protein or JAK/STAT signaling pathways that regulate cell growth, survival, proliferation and chemotaxis (Busillo and Benovic, 2007; Miller et al., 2008).

The atypical receptor ACKR3 (also known as CXCR7) regulates CXCL12 gradients through a scavenging mechanism which promotes the migration of CXCR4-expressing cells along these gradients. Dysregulation of the CXCR4/CXCL12 signaling axis has been implicated in various pathological conditions, including cancer (Balkwill, 2004; Chatterjee et al., 2014; Buck et al., 2022), viral infections (Bleul et al., 1996; Britton et al., 2021), cardiovascular diseases (Liehn et al., 2011; Hyafil et al., 2017; Jo et al., 2025), autoimmune disorders (Balabanian et al., 2005), pulmonary fibrosis (Jaffar et al., 2020), among others. CXCR4 was first identified as a co-receptor for CD4^+^ T-cell tropic human immunodeficiency virus 1 (HIV-1), facilitating entry by binding to the gp120 envelop glycoprotein. Subsequent studies demonstrated that the viral macrophage inflammatory protein II (vMIPII), encoded by the Kaposi’s sarcoma-associated herpesvirus, inhibits multiple chemokines including CXCR4 (LiWang et al., 1999). These studies provided initial structural insights and a model for the design of anti-HIV therapeutics targeting CXCR4. In cancer, CXCR4 is overexpressed in multiple tumor types and is involved in mechanisms associated with cancer aggressiveness, such as tumor growth (Rubin et al., 2003; Smith et al., 2004), metastasis (Karaman and Detmar, 2014; Kim et al., 2010) and recruitment of immunosuppressive leukocytes (Chen et al., 2015; Fortunato et al., 2020). AMD3100 (Plerixafor) and BL-8040 (Motixafortide) received U.S. Food and Drug Administration (FDA) approval for hematopoietic stem cell mobilization in combination with G-CSF for autologous transplantation in 2008 and 2023, respectively. However, inhibition of the CXCR4:CXCL12 axis for chemosensitization and its antitumor efficacy remains under investigation (Ghobrial et al., 2019; Borthakur et al., 2021; Martin et al., 2021; Andtbacka et al., 2022).

**FIGURE 1:**
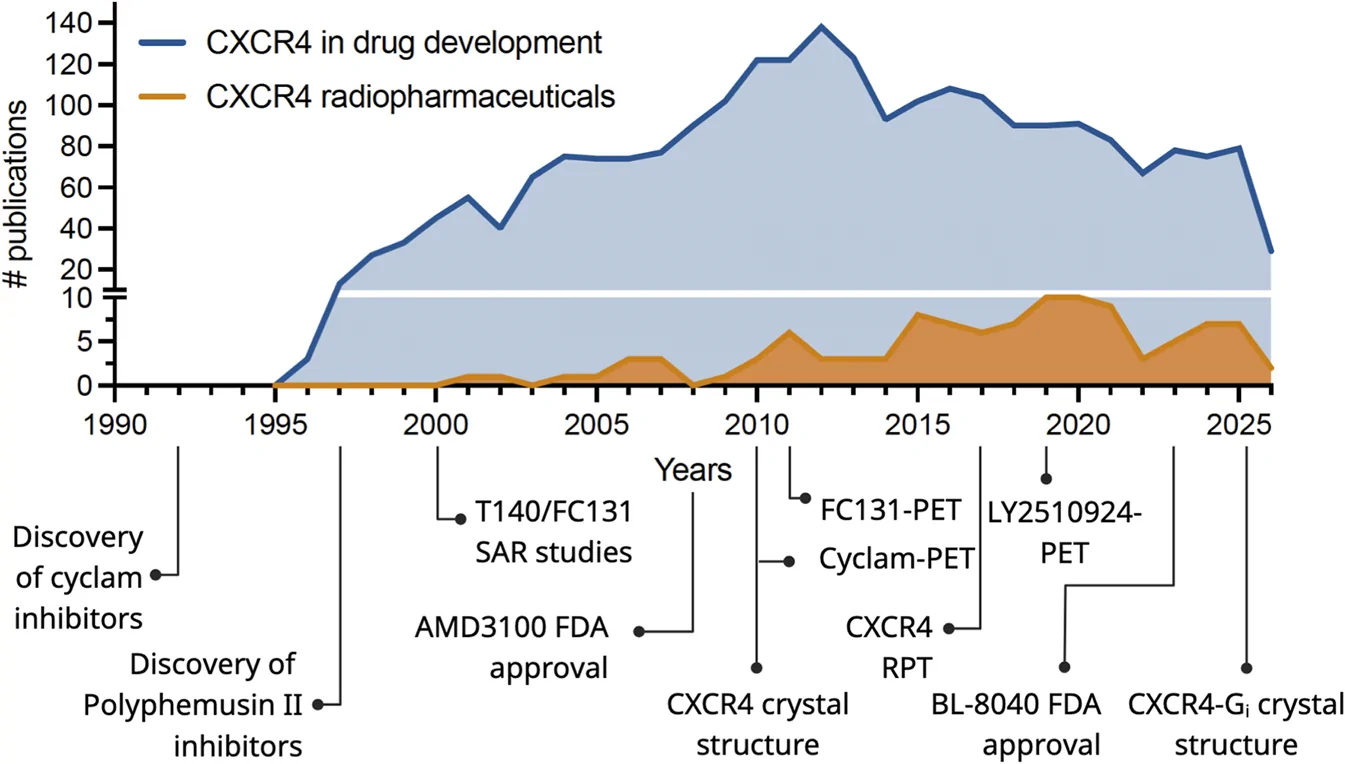
Number of publications related to CXCR4 drug development and radiopharmaceuticals listed in the PubMed database. The figure indicates the increase in drug development targeting CXCR4 (blue) in the late 1990s and the FDA approval of two CXCR4 inhibitors in 2008 and 2023. Publications related to CXCR4 radiopharmaceuticals in the drug development space (orange) had a strong upward trend in publications in the past 10 years. Search terms used are shown in Supplementary Material.

CXCR4 has long been established as a relevant therapeutic target, but it is only within the last 2 decades that its potential for radiopharmaceutical development has been investigated (Figure 1). Most radiopharmaceuticals are derived from natural ligands or previously published high-affinity inhibitors and subsequently developed as molecular imaging agents. However, the number of compounds that have seen translation to therapeutic agents remains limited. Radiotheranostics are drugs that combine a targeting molecule with a radioisotope to enable molecular imaging or targeted radiopharmaceutical therapy (RPT) (Sgouros et al., 2020; Kuten et al., 2026). The targeting moiety, which can be a small molecule, peptide or antibody, is designed to bind with high affinity and selectivity to a receptor for the targeted delivery of radiation. Based on the physical decay properties of the radioisotope, these drugs can be used for imaging by positron emission tomography (PET) (e.g., ^11^C, ^18^F, ^68^Ga, ^64^Cu) or single photon emission computed tomography (SPECT) (^99m^Tc, ^111^In), or for RPT using either beta-particle emitter (e.g., ^177^Lu, ^90^Y, ^161^Tb, ^67^Cu, ^203^Pb) or alpha-particle emitter (e.g., ^225^Ac, ^221^At, ^212^Pb) (Kostelnik and Orvig, 2019). Multiple strategies have been developed to facilitate stable radiolabeling with different radioisotopes, including direct substitution, the use of novel prosthetic groups or bifunctional chelators, and *in vivo* pre-targeting approaches (McBride et al., 2012; Meyer et al., 2016). Successful development of radiotheranostics requires careful selection of an overexpressed receptor, optimization of the targeting vector’s pharmacokinetic and metabolic properties, and appropriate matching of the radioisotope’s physical decay characteristics.

CXCR4 inhibitors and chemokine mimetics are well characterized scaffolds for the design and development of drug conjugates and radiopharmaceuticals (Gourni et al., 2011; Costa et al., 2019; Lau et al., 2019; Osl et al., 2020). Optimization of the pharmacophore, linker, prosthetic group or bifunctional chelator is essential for improving binding affinity, *in vivo* stability and pharmacokinetic profile. The success of a radiotheranostic depends on rapid clearance from normal organs and prolonged tumor retention to minimize radiation-associated toxicities while maximizing imaging contrast and therapeutic efficacy. Although many CXCR4-targeting radiopharmaceuticals have been developed, the majority remain at the preclinical stage due to poor tumor retention or undesirable uptake in normal organs. A limited number of promising CXCR4 PET imaging compounds have been evaluated in patients; however, formal clinical trials are required to establish their clinical utility and benefit over alternative approaches. For example, the peptide-based theranostic pair, Pentixafor/Pentixather, has advanced to early clinical evaluation for CXCR4 RPT under compassionate use for critically ill patients (Lapa et al., 2017a). Ongoing preclinical studies continue to explore novel ligands, linker compositions, and chelation strategies to enhance tumor targeting for the development of CXCR4-directed theranostics. Recent literature has provided a comprehensive overview regarding the clinical use of the Pentixafor/Pentixather radiotheranostic pair and other emerging scaffolds (Yu et al., 2023; Yang et al., 2026). Although recent derivatives have shown promising results as radiotheranostics, a knowledge gap remains in translating structural insights into drug design, applications of emerging radioisotopes and addressing major toxicity concerns. This review summarizes recent CXCR4 structural data and provides a comparative analysis of chemical modifications and application of novel radioisotopes that led to the development of improved small molecule and peptide-based radiopharmaceuticals for imaging and/or therapy.

## 2 CXCR4 structure and molecular basis of ligand recognition

**FIGURE 2:**
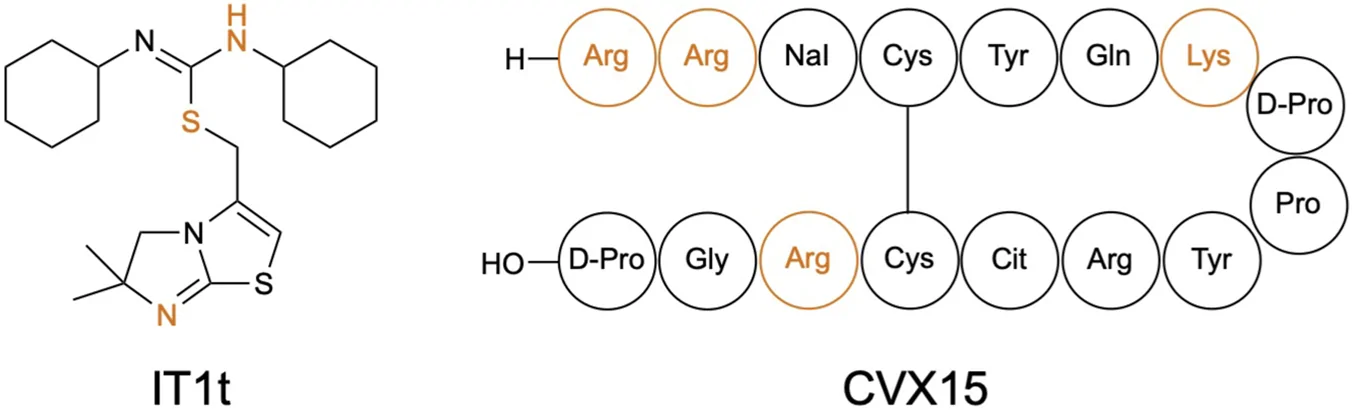
Structures of example CXCR4 small molecule and peptide inhibitors. Atoms of IT1t and residues of CVX15 involved in ligand interactions with CXCR4 are shown in orange.

Structural insights into CXCR4 interactions with CXCL12, small molecules, peptides and antibodies provide valuable information for the design of novel ligands and guide chemical modifications. Currently, 29 structures of CXCR4 are available in the Protein Data Bank (PDB), including both active and inactive conformational states (Table 1). CXCR4 adopts the canonical seven transmembrane architecture characteristic of GPCRs, consisting of an unresolved flexible extracellular N-terminus, three intracellular loops, and three extracellular loops (ECL1, ECL2, ECL3). Compared to the inactive state, crystal structures of the active state showed an outward positioning of helix VI at the intracellular region facilitating G protein association and activation. Moreover, it resulted in structural changes of the binding pocket (Saotome et al., 2025). The first structures were obtained by X-ray crystallography methods of CXCR4 as a homodimer in its inactive state. The authors reported the crystal structure of CXCR4 homodimers in complex with a small molecule (IT1t) and cyclic peptide (CVX15) inhibitors with resolutions of 2.5 Å and 2.9 Å, respectively (Wu et al., 2010). Both ligands occupied the orthosteric binding pocket (Chemokine Recognition Site 2; CRS2) interacting with negatively charged aspartic acid and glutamic acid residues (Figure 2). IT1t was positioned in the minor subpocket and formed interactions through its nitrogen and sulfur atoms with D97, D187 and E288. In contrast, CVX15 occupied most of the shallow extracellular major subpocket and resulted in interaction mainly through the arginine residues with D171, D187 and D262 while the side-chain of NaI^3^ was positioned in a hydrophobic region close to helix V. Among these residues, E288 is at the boundary between the minor and major subpockets of the binding site and has been previously reported to be essential for CXCL12 binding and HIV-1 recognition (Tian et al., 2005).

**TABLE 1:** Summary of available CXCR4 structures in the Protein Data Bank (PDB) database.

PDB ID	Ligand and stoichiometry	Method	Resolution	Form	Status	Protein modifications	References
3OE0	CVX15 (1:1)	X-ray	2.90 Å	Dimer	Inactive	T4L	(Wu et al., 2010)
3ODU	IT1t (1:1)	X-ray	2.50 Å	Dimer	Inactive	T4L	(Wu et al., 2010)
4RWS	vMIP-II (1:1)	X-ray	3.10 Å	Dimer	Inactive	Disulfide bonds	(Qin et al., 2015)
8U4P	AMD3100 (1:1)	Cryo-EM	3.15 Å	Monomer	Active (G_i_-bound)	FLAG tag	(Saotome et al., 2025)
8U4O	CXCL12 (1:1)	Cryo-EM	3.29 Å	Monomer	Active (G_i_-bound)	FLAG tag	(Saotome et al., 2025)
8U4N	Apo	Cryo-EM	2.72 Å	Monomer	Active (G_i_-bound)	FLAG tag	(Saotome et al., 2025)
8U4R	REGN7663 (1:1)	Cryo-EM	3.10 Å	Monomer	Active (G_i_-bound)	FLAG tag	(Saotome et al., 2025)
8K3Z	CXCL12 (1:1)	Cryo-EM	2.81 Å	Monomer	Active (G_i_-bound)	scFv16	(Liu et al., 2024)
9MDU	Apo	Cryo-EM	2.90 Å	Tetramer	Inactive	FLAG tag	(Zhang et al., 2025)
9MEJ	gp120 (1:1)	Cryo-EM	3.99 Å	Monomer	Inactive	FLAG tag	(Zhang et al., 2025)
9MEU	CXCL12 (2:1)	Cryo-EM	3.46 Å	Tetramer	Inactive	FLAG tag	(Zhang et al., 2025)
9ME1	CXCL12 (1:1)	Cryo-EM	3.37 Å	Octamer	Inactive	FLAG tag	(Zhang et al., 2025)
8ZPL	HF51116 (1:1)	Cryo-EM	3.01 Å	Monomer	Inactive	BRIL, L125W, T240P, kOR-Nb6	(Sang et al., 2025)
8ZPM	AMD070 (1:1)	Cryo-EM	3.20 Å	Monomer	Inactive	BRIL, L125W, T240P, kOR-Nb6	(Sang et al., 2025)
8ZPN	AMD3100 (1:1)	Cryo-EM	3.31 Å	Monomer	Inactive	BRIL, L125W, T240P, kOR-Nb6	(Sang et al., 2025)
8YU7	Apo	Cryo-EM	3.01 Å	Tetramer	Inactive	FLAG tag	(Liu et al., 2025)

**FIGURE 3:**
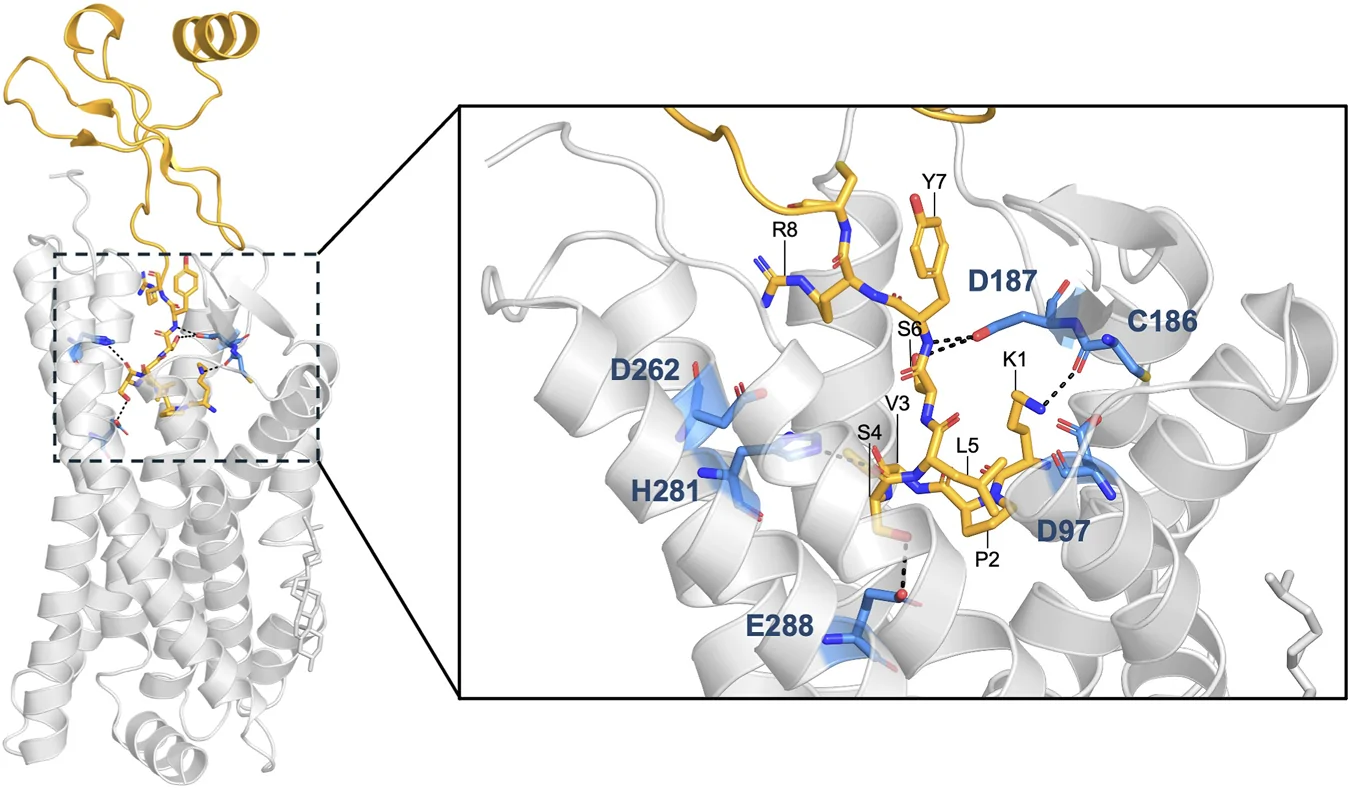
Example of the crystal structure of the CXCR4:CXCL12 complex in the active state (PDB ID: 8U4O) (Saotome et al., 2025). Polar interactions (dashed lines) between CXCL12 N-terminus (orange) and residues within CXCR4 CRS2 (blue) are depicted. Structure was generated using PyMOL Molecular Graphics System, Version 3.2.0a Schrödinger, LLC.

The structure of CXCR4 in active state was recently resolved by cryo-electron microscopy in complex with G_i_ proteins and showed a 1:1 stoichiometry for the CXCL12 and AMD3100 complexes (Liu et al., 2024; Saotome et al., 2025). The three active structures showed an overall conserved architecture. CXCL12 binding induced only minimal conformational changes compared to the unbound and AMD3100-bound CXCR4 structures characterized by a rotation of E288 and a 0.7–1 Å outward movement of helix VII (Saotome et al., 2025). CXCL12 binds to CXCR4 using a 2-site binding model typical of chemokine:GPCR interactions. The N-terminus of CXCL12 is positioned horizontally into the minor pocket of the receptor’s binding site and adopts a vertical position toward the extracellular space after residue S4. CXCL12 showed interactions with residues in ECL2 (D97, C186, D187) and helix VII (H281, E288) (Figure 3) (Liu et al., 2024; Saotome et al., 2025). These studies support previous evidence showing that truncation of the N-terminus of CXCL12 (NH_2_-KPV) inhibited CXCR4 binding and activation whereas C-terminal truncation only had a reduced effect (Crump et al., 1997; Janssens et al., 2018). Moreover, Liu et al. validated a polar interaction between the residue C28 at the CRS1.5 of CXCR4 and the P10 residue of CXCL12 (“X” position of the CXC motif) by site-directed mutagenesis analysis. This suggests the possible selectivity of CXCR4 towards CXCL12. However, interactions of the globular domain with the flexible CXCR4 N-terminus have not been fully resolved.

**FIGURE 4:**
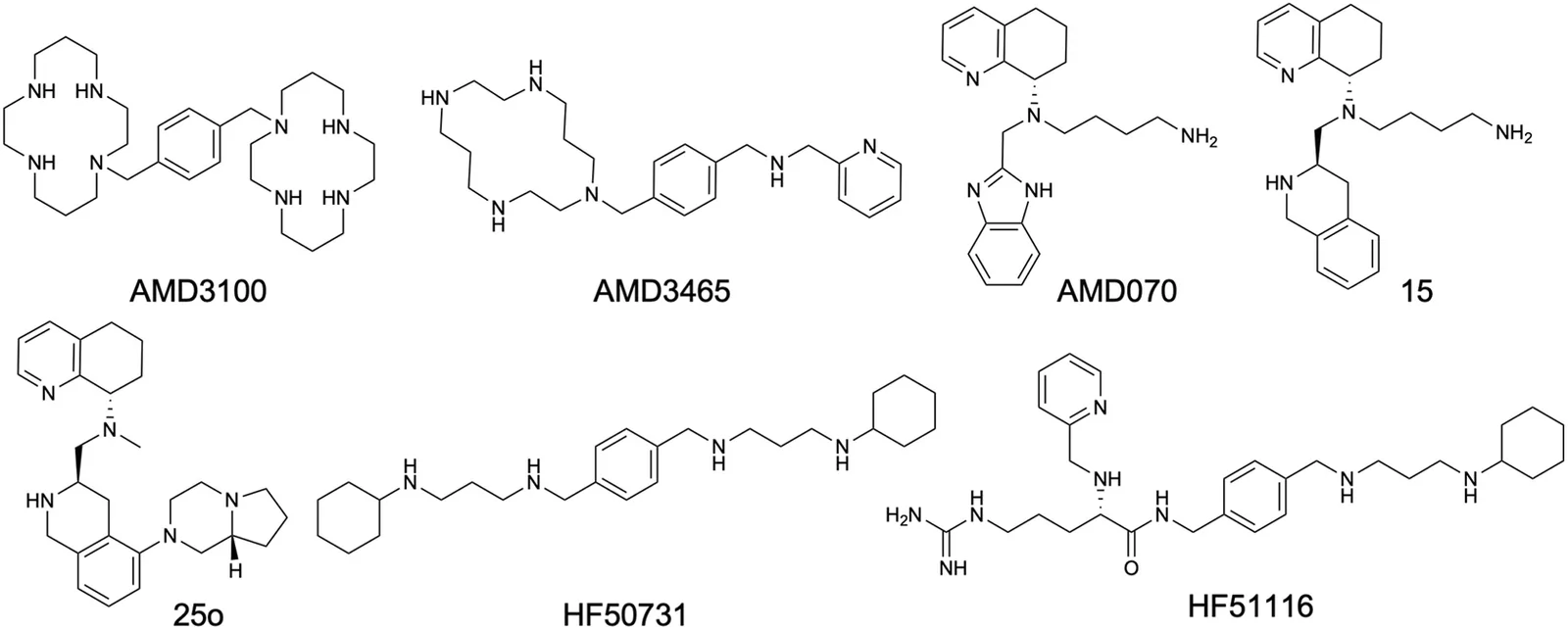
Structure of selected small organic CXCR4 inhibitors.

Complexation with three different inhibitors (AMD3100, AMD070 and HF51116) revealed overlapping interactions with residues involved in CXCL12 binding. Example structures are shown in Figure 4. These compounds interact with residues from both the major and minor subpockets of CXCR4. Site-directed mutagenesis studies showed that Y45, W94, Y255, D262 and E288 are critical for AMD3100 binding, while HF51116 binding is mediated through interactions with W94, D97, D262 and E288 (Sang et al., 2025). The AMD3100-CXCR4 cryo-EM structure shows that AMD3100 adopts a diagonal orientation within the transmembrane cavity, where the cyclam rings interact with D262 and E288 through electrostatic interactions and the aromatic linker region may have hydrophobic contacts with I284. Interestingly, the AMD3100 inhibitor binds to the active CXCR4-G_i_ complex without disrupting receptor-G protein coupling, suggesting that AMD3100 may function as a partial agonist or modulator of active CXCR4 (Saotome et al., 2025). These results are consistent with previous CXCR4 mutagenesis studies identifying D171 and D262 as essential residues involved in AMD3100 binding and inhibitory activity (Gerlach et al., 2001; Hatse et al., 2001). In contrast to small molecule and peptide inhibitors, the fully human anti-CXCR4 monoclonal antibody REGN7663 inhibits receptor activation by steric blockade of CXCL12 engagement. REGN7663 exhibited potent inhibitory activity in a competition assay (IC_50_: 2.7 ± 0.1 nM) and inverse agonist activity (EC_50_: 1.3 ± 0.4 nM) measured using a cyclic adenosine monophosphate response element luciferase assay. Most of the residues from the REGN7663 paratope interact with residues of the ECL2 and N-terminus while the CDR-H3 loop partially inserts in the binding cavity interacting with the residue E288 through R105.

Experimentally determined CXCR4 structures in complex with different ligands provide a template that can be explored for the development of radiotheranostics, alternative drug conjugates or novel scaffolds. The receptor´s orthosteric site is capable of accommodating ligands across a wide range of molecular sizes. Although the analyzed structures in this section show that diverse ligands bind to different subpockets, these interactions are commonly with hydrophobic and negatively charged residues (Supplementary Figure S1). Crystal structures obtained through different protein stabilization approaches showed that E288 is a conserved residue shared across all ligands and D262 or D97 are conserved only among a subset of ligands. These structures provide a molecular foundation for potential anchor points for the design of novel scaffolds. Beyond ligand recognition, recent structural studies have shown diverse states of CXCR4 organization within the cellular membrane. Saotome et al. reported the formation of CXCR4 homomeric trimers and tetramers that assemble into higher-order “superclusters” containing a lipid-associated cavity along the central axis (Liu et al., 2025). Comparison of the protomers with previously reported structures of inactive and active CXCR4 showed an allosteric antagonist state. Regulatory mechanisms of CXCR4 oligomers and their implications in drug targeting remain to be investigated.

## 3 Small molecule-based CXCR4 radiopharmaceuticals

### 3.1 CXCR4 inhibitors

Different classes of small organic molecules have been developed as CXCR4 inhibitors (Figure 4). AMD3100 was initially developed as an inhibitor of HIV infection in CD4^+^ T cells (Donzella et al., 1998; De Clercq, 2003). During clinical evaluation, AMD3100 unexpectedly mobilized CD34^+^ stem and progenitor cells through selective inhibition of the CXCL12-CXCR4 axis. These findings motivated the clinical application of AMD3100 for stem cell mobilization prior to transplantation (Hendrix et al., 2000; Liles et al., 2003; DiPersio et al., 2007). AMD3100 is a symmetrical bicyclam, however, SAR studies identified a non-symmetrical CXCR4 inhibitor, providing the foundation for the development of next-generation CXCR4 antagonists. The monocyclam AMD3465 was designed by removing one cyclam ring to reduce the overall positive charge at physiological pH while maintaining the nitrogen atoms essential for its inhibitory potency (Hatse et al., 2005; Bridger et al., 2010). This work inspired the design of an optimized non-macrocycle CXCR4 inhibitor, AMD070, incorporating tetrahydroquinoline (THQ), benzimidazole and butylamine containing scaffolds (Skerlj et al., 2010). Incorporation of THQ groups linked to pyridine or imidazole derivatives improved antiviral activity, whereas replacement with phenyl or primary amine groups significantly reduced their potency. Further optimization through substitution of the benzimidazole moiety with a tetrahydroisoquinoline (TIQ) resulted in potent CXCR4 inhibitors with improved bioavailability (compounds 15o and 25o) (Truax et al., 2013; Nguyen et al., 2018). More recently, polyamine-based CXCR4 inhibitors have been developed using fragment-based combinatorial drug approaches (Fang et al., 2020; 2022). HF50731 was designed to contain a phenylenebis(methylene) linker and two [3-(cyclohexylamino)propyl]amino moieties in para position (Fang et al., 2020). The authors demonstrated the importance of a small aromatic ring system and its orientation through SAR studies. An asymmetrical polyamine, HF51116, was designed by integrating four validated CXCR4 pharmacophores resulting in high binding inhibition and potent stem cell mobilization (Fang et al., 2021; 2022). Site-directed mutagenesis and crystallography studies further identified W94, D97, D171, D262 and E288 as residues essential for CXCR4 binding. Several radiopharmaceuticals have been developed based on the previously described scaffolds. Most radiopharmaceuticals within this class have been labeled with positron-emitting radioisotopes (^64^Cu, ^68^Ga and ^18^F) (Table 2). However, their translation into therapeutic radioligands has been limited primarily due to high uptake in normal organs.

**TABLE 2:** Selected small molecule-based CXCR4 radiopharmaceuticals. The compound described is the lead compound selected for the study. Tumor-to-organ ratios for muscle (T/M), blood (T/B), kidneys (T/K) and liver (T/L). Uptake values are represented as % of injected activity per Gram of tissue (%IA/g) or mean standardized uptake values (SUV_mean_).

Compound	Scaffold and modifications	Binding properties^a^	Physicochemical properties	*In vivo* biodistribution^b^	References
[^64^Cu]Cu-AMD3100	Bicyclam	^*125*^ *I-SDF-1α competition, IC*_*50*_ *:* 84 nM (CHO:hCXCR4 cells, 0.07 nM) *Chemotaxis:* 75.4 nM (Jurkat cells) *AMD3100 competition, IC*_*50*_ *:* 62.7 µM (Jurkat cells), 47.0 µM (Mouse splenocytes)	RCP: >98% LogD_7.4_: 0.52 ± 0.02 MA: ND	DU4475 (Breast cancer) 90 min p.i.: 26.2 ± 3.5 (T/M) U87-stb-CXCR4 (Engineered cell line) 90 min p.i.: 16.9 (T/B) U87 (GBM, 90 min p.i.): 2.6 (T/B)	(Jacobson et al., 2009; Nimmagadda et al., 2010; Weiss et al., 2012)
[^64^Cu]Cu-AMD3465	Monocyclam	ND	RCP: >98% LogD_7.4_: −2.71 ± 0.37 MA: 6.0 ± 3.1 MBq/nmol	U87-stb-CXCR4 (Engineered cell line) 90 min p.i.: 96.3 ± 14.0 %IA/g (T), 2.9 (T/L), >100 (T/B), 2.5 (T/K) U87 (GBM) 90 min p.i.: 4.2 ± 0.9 %IA/g (T), 0.1 (T/L), 6.4 (T/B), 0.1 (T/K)	(De Silva et al., 2011)
[^68^Ga]Ga-11	Bicyclam -PEG linker and NODAGA chelator	^*125*^ *I-SDF-1α competition, IC*_*50*_ *:* 122 nM (Jurkat cells)	RCP: >92% LogD_7.4_: −1.68 MA: 17 MBq/nmol	H69 (SCLC) 60 min p.i.: 7.06 (T/M), 0.02 (T/L)	(Poty et al., 2016)
[^18^F]F-RPS-544	Monocyclam -Fluoropyridine	^*68*^ *Ga-CPCR4 competition, IC*_*50*_: 6.3 ± 0.8 nM (PC3-CXCR4 cells) *Saturation assay, K*_*D*_: 5.1 ± 1.0 nM (PC3-CXCR4)	RCP: >99% LogD_7.4_: ND MA: >185 MBq/nmol	PC3-CXCR4 (Engineered cell line) 60 min p.i.: 3.4 ± 1.2 %IA/g, 2.5 (T/B), 0.1 (T/L), 0.1 (T/K)	(Amor-Coarasa et al., 2018, Amor-Coarasa et al., 2019)
[^18^F]F-RPS-547	Monocyclam -Fluoroethyl triazole	^*68*^ *Ga-CPCR4 competition, IC*_*50*_: 601 ± 118 nM (PC3-CXCR4 cells, 100 nM) *Saturation assay, K*_*D*_: 261 ± 22 nM (PC3-CXCR4)	RCP: >97% LogD_7.4_: ND MA: >185 MBq/nmol	PC3-CXCR4 (Engineered cell line) 60 min p.i.: 3.1 ± 0.5 %IA/g, 10.1 (T/B), 0.5 (T/L), 0.3 (T/K)	(Amor-Coarasa et al., 2019)
[^18^F]F-RPS-534	Monocyclam -Fluoroethyl triazole	^*68*^ *Ga-CPCR4 competition, IC*_*50*_: 218 ± 38 nM (PC3-CXCR4 cells, 100 nM) *Saturation assay, K*_*D*_: 176 ± 30 nM (PC3-CXCR4)	RCP: >97% LogD_7.4_: ND MA: >185 MBq/nmol	PC3-CXCR4 (Engineered cell line) 60 min p.i.): 7.2 ± 0.3 %IA/g (T), 27.5 (T/B). 0.4 (T/L), 1.1 (T/K)	(Amor-Coarasa et al., 2019)
[^18^F]F-MCFB	Monocyclam -FBA	^*125*^ *I-SDF-1α competition, IC*_*50*_ *:* 111.3 nM (U87.CD4.CXCR4 cells, 0.1 nM)	RCP: ND LogD_7.4_: −1.7 MA: 5.7 MBq/nmol	U2932 (Lymphoma) 60 min p.i.: 3.3 ± 0.91 %IA/g (T), 0.05 (T/L)	(Brickute et al., 2019)
[^68^Ga]Ga-DO3A-PEG-Ni_2_-AMD3100	Bicyclam -PEG linker and DOTA chelator	^*125*^ *I-SDF-1α competition, IC*_*50*_ *:* 516 nM (H69 cells)	RCP: ND LogD_7.4_: −3.66 ± 0.03 MA: 2.4 ± 1.5 MBq/nmol	U87-CXCR4 (Engineered cell line) 60 min p.i.: 3.3 ± 0.6 %IA/g (T), 0.09 (T/L)	(Renard et al., 2023)
[^18^F]F-SFB-AMD3465	Monocyclam -SFB	*Homologous displacement, IC*_*50*_: 67.2 ± 7.2 nM (4T1 cells)	RCP: >98% LogD_7.4_: −0.91 ± 0.08 MA: >120 MBq/nmol	4T1 (Murine breast cancer) 120 min p.i.: 2.34 ± 0.15 %IA/g (T), 2.5 (T/B), T/K (0.2)	(Li et al., 2024)
[^68^Ga]Ga-TD-01	AMD070 (THQ/TIQ) -DOTA chelator	^*125*^ *I-SDF-1α competition, IC*_*50*_ *:* 36.5 nM (Jurkat cells, 2.9 nM)	RCP: >99% LogD_7.4_: −1.1 MA: 55.8 ± 2.0	GL261 orthotopic model	(Suwattananuruk et al., 2024)
[^68^Ga]Ga-SDNUM04	Aniline	*Saturation assay, K*_*D*_: 32.2 ± 0.7 nM (HT-29)	RCP: >99% LogD_7.4_: −2.9 MA: 59.2 ± 2.1 MBq/nmol	HT-29 (Colorectal adenocarcinoma) 45 min p.i.): 4.3 ± 0.4 %IA/g (T), 1.07 (T/L) Daudi (Lymphoma) 45 min p.i., SUV_mean)_: 0.6 ± 0.1 (T), >1.5 (T/L)	(Wang et al., 2025)

^a^ Values reported as half-maximal inhibitory concentration (IC_50_) are only directly comparable when determined under the same experimental conditions. The cell line and concentration of the labeled ligand are indicated when available.

^b^ Tumor uptake and tumor-to-organ ratios are reported for different tumor models that may exhibit varying levels of CXCR4 expression.

RCP, Radiochemical purity; MA, Molar activity; T, Tumor uptake; GBM, Glioblastoma; SCLC, Small cell lung carcinoma; ND, Not disclosed.

### 3.2 Cyclam derivatives

**FIGURE 5:**
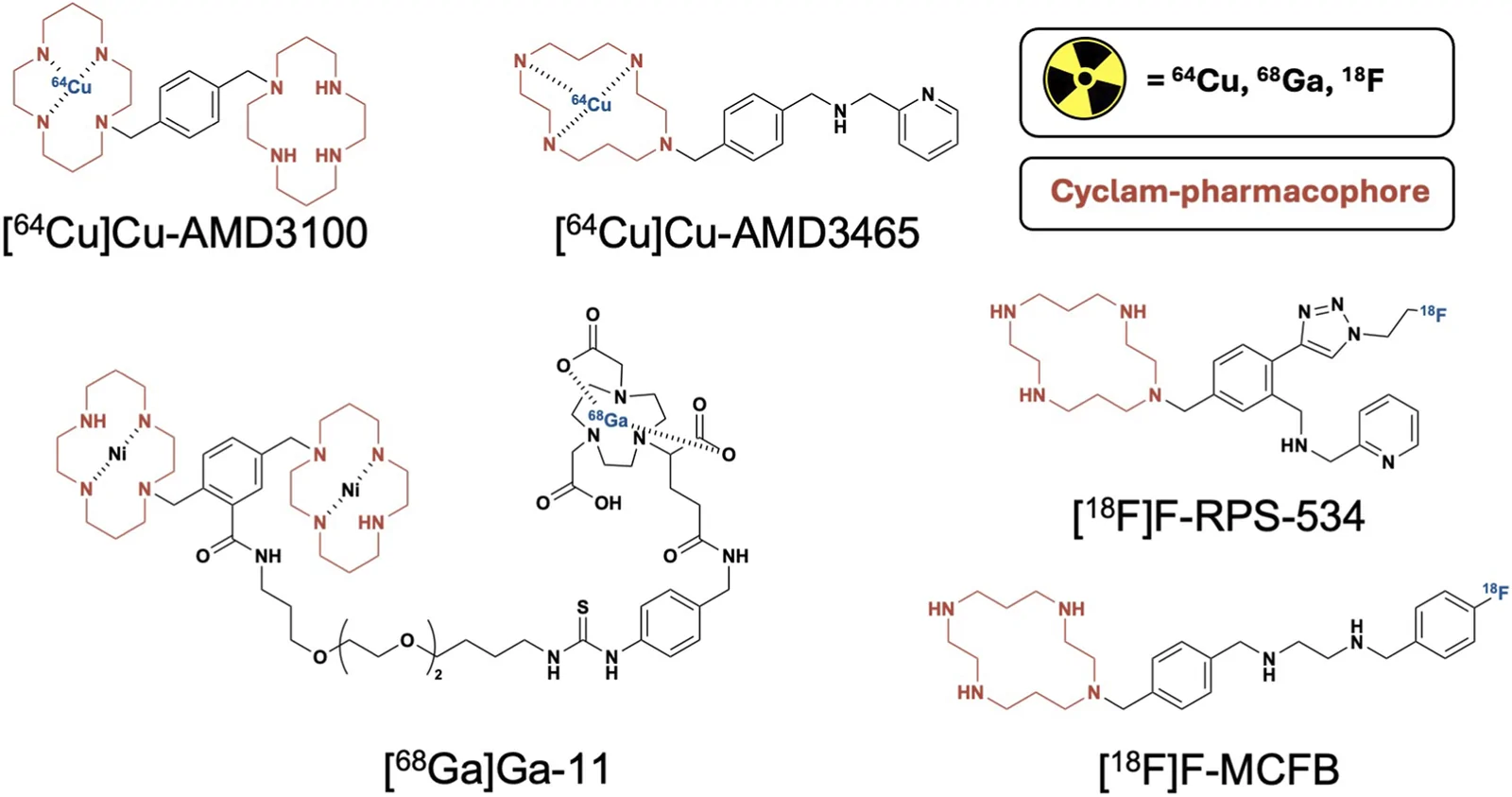
Examples of cyclam-derived radiopharmaceuticals labeled with ^64^Cu, ^68^Ga or ^18^F.

The bicyclam AMD3100 was among the first scaffolds used for CXCR4 PET imaging (Figure 5). AMD3100 analogues coordinated with transition metal ions (Cu^2+^, Zn^2+^ and Ni^2+^) demonstrated enhanced binding affinity through interactions with D262 residue in the CXCR4 binding pocket (Gerlach et al., 2003). These studies supported its use as a scaffold for radiopharmaceutical development. AMD3100 was directly labeled with ^64^Cu with high radiochemical purity (RCP, >98%) and showed comparable inhibition of SDF-1α-mediated migration compared to the precursor (IC_50_ = 75.4 and 27.5 nM, respectively). Also, it exhibited similar binding properties to both mouse and human CXCR4 (Jacobson et al., 2009). *In vivo* biodistribution studies in healthy immunocompetent mice revealed high accumulation in CXCR4-expressing tissues including the spleen, bone marrow, lymph nodes, kidneys and liver. Co-injection with non-radioactive AMD3100 or SDF-1α reduced uptake in liver and lymphoid organs indicating CXCR4-mediated uptake. [^64^Cu]Cu-AMD3100 demonstrated specific tumor uptake across different tumor models which correlated directly with receptor density (U87-stb-CXCR4 > DU4475 > MDA-MB-231 > U87). Although favorable tumor-to-blood and tumor-to-muscle uptake ratios were observed, it showed significant liver uptake (>40% IA/g) (Nimmagadda et al., 2010; Weiss et al., 2012; Muz et al., 2020). In an independent study, Waldenström Macroglobulinemia cells that were cultured under hypoxic conditions showed increased CXCR4 expression and higher *in vivo* tumor uptake of [^64^Cu]Cu-AMD3100 (Muz et al., 2020). Radiolabeling of the monocyclam derivative AMD3465 with ^64^Cu showed improved hydrophilicity and higher tumor-to-blood ratios compared to [^64^Cu]Cu-AMD3100 (De Silva et al., 2011). However, liver uptake remained high (>30 %IA/g) resulting in a suboptimal biodistribution profile. Liver uptake was attributed to both CXCR4 expression in the liver and the *in vivo* instability of the cyclam:Cu(II) complex.

Cyclam analogues were modified to incorporate prosthetic groups or chelators for radiolabeling with ^18^F and ^68^Ga isotopes while improving metabolic stability and reducing non-specific liver uptake. A series of bis-nickel AMD3100 analogues that contain a polyethylene glycol (PEG) linker with three repeating units coupled to a 1,4,7,10-tetraazacyclododecane-1,4,7,10-tetraacetic acid (DOTA), 1,4,7-triazacyclononane,1-glutaric acid-4,7-diacetic acid (NODAGA) or p-NCS-Benzyl-NODAGA (compound 11) chelators were synthesized and radiolabeled with ^68^Ga. All derivatives showed reduced CXCR4 binding affinity (9 to 106-fold) compared to AMD3100. ^68^Ga-labeling resulted in poor RCP and only compound 11 was labeled at >92% RCP after purification. Despite [^68^Ga]Ga-11 exhibiting improved hydrophilicity compared to [^64^Cu]Cu-AMD3100 (LogD_7.4_ = −1.50 ± 0.18 vs. 0.52 ± 0.02), liver uptake remained high, with poor tumor-to-organ ratios in an H69 tumor model (Poty et al., 2016). Subsequent modifications incorporating PEG linkers and a DOTA chelator achieved partial reduction in liver uptake (Renard et al., 2023).

Alternative strategies focused on adapting monocyclam AMD3465 for ^18^F-labeling. The Babich group synthesized a series of AMD3465 analogues using fluoropyridine and fluoroethyltriazole motifs as radiolabeling handles. [^18^F]F-RPS-544 exhibited high *in vitro* binding affinity but biodistribution studies resulted in poor biodistribution and *in vivo* defluorination (Amor-Coarasa et al., 2018). [^18^F]F-RPS-534 and [^18^F]F-RPS-547 were later identified from an *in silico* library screening of fluorine-containing derivatives. These fluoroethyltriazol-derived compounds showed improved tumor-to-organ ratios compared to [^18^F]F-RPS-544 and were cleared mainly *via* the renal pathway despite their high liver uptake. The authors found inconsistencies between the obtained docking scores and the *in vitro* inhibitory constants suggesting possible secondary interactions with other chemokine receptors and highlighting differences in binding to murine and human CXCR4. [^18^F]F-SFB-AMD3465 and [^18^F]F-MCFB were synthesized using a fluorobenzene prosthetic group (Brickute et al., 2019; Li et al., 2024). [^18^F]F-MCFB was synthesized using a 4-[^18^F]-fluorobenzaldehyde prosthetic group to retain the same number of nitrogen atoms as the reference compound AMD3465 and preserve binding affinity (Brickute et al., 2019). [^18^F]F-MCFB demonstrated good metabolic stability in plasma and liver homogenates for at least 60 min. However, no reduction in liver uptake was observed *in vivo*. Pre-treatment with metformin (50 mg/kg, 30 min prior to injection) showed a slight increase in tumor uptake and decrease in kidneys and liver uptake; suggesting that the expression of organic cation transporters in the hepatobiliary system may partially contribute to the observed liver uptake (Kusuhara and Sugiyama, 2010).

### 3.3 Other small organic derivatives

**FIGURE 6:**
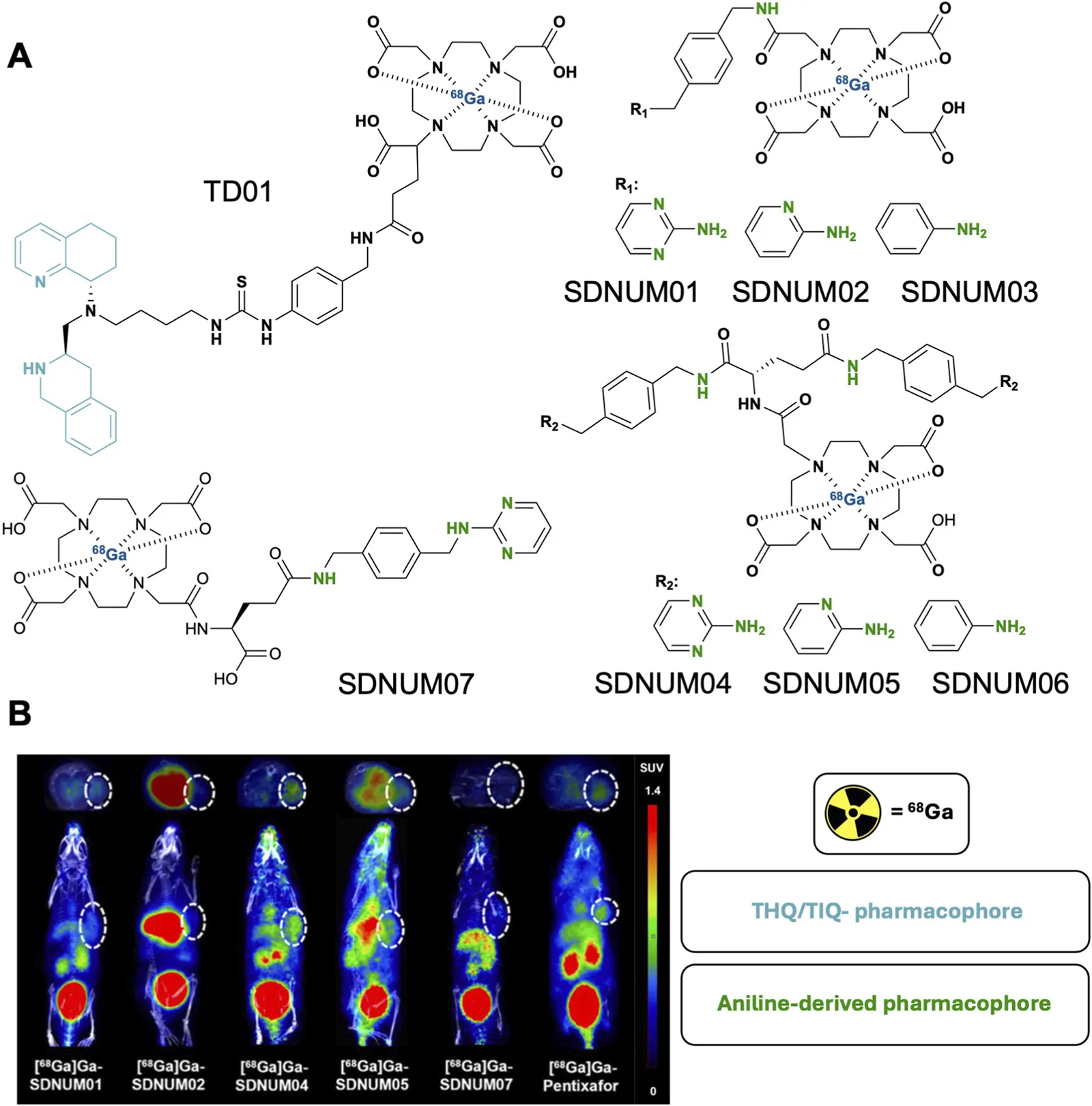
**(A)** Examples of novel small organic CXCR4-targeting radiopharmaceuticals. THQ: tetrahydroquinoline, TIQ: tetrahydroisoquinoline. **(B)** PET/CT images of ^68^Ga-labeled SDNUM01, SDNUM02, SDNUM04, SDNUM05, SDNUM07 and Pentixafor in mice engrafted with HT29 tumors at 45 min p.i. Scale bar is shown as mean standardized uptake value (SUV_mean_). Adapted with permission from (Wang et al., 2025). Copyright 2025 American Chemical Society.

More recently, novel nitrogen-based small organic radiopharmaceuticals have been synthesized and evaluated in preclinical tumor models (Figure 6A). A benzenesulfonamine-based radioligand labeled with ^18^F was designed using predictive quantitative structure-activity relationship modeling (Oum et al., 2020). The lead compound, [^18^F]F-5 was evaluated *in vivo* in two different tumor xenograft models. [^18^F]F-5 resulted in 3.3-fold and 1.3-fold tumor-to-liver ratios in a squamous cell carcinoma of head and neck orthotopic model and lung metastatic tumor model at 90 min p.i., respectively. TD01 was designed using a THQ/TIQ scaffold and a DOTA chelator. However, PET images showed high uptake in liver, intestine and kidneys (Suwattananuruk et al., 2024). Recently a novel class of small organic radioligands was reported using three different aniline derivative-benzylamine groups. Monomeric and dimeric derivatives were synthesized for *in vitro* evaluation. These compounds showed superior binding affinity and stability compared to compounds containing two or three nitrogen atoms within the targeting moiety (Wang et al., 2025). All compounds were radiolabeled with ^68^Ga with high radiochemical purity (>95%) and molar activity of 44.4–59.2 MBq/nmol (Figure 6B). A dimeric candidate, [^68^Ga]Ga-SDNUM04, had lower liver uptake and favorable tumor-to-liver ratios (T/L > 1). A head-to-head comparison with the cyclic peptide [^68^Ga]Ga-Pentixafor showed similar tumor uptake (4.25 ± 0.35 vs. 3.65 ± 0.08 %IA/g) and tumor-to-organ ratios at 45 min after injection in HT-29 tumor-bearing mice. Cross-reactivity of [^68^Ga]Ga-SDNUM04 was confirmed in a murine tumor model (4T1), however, uptake in organs with physiological CXCR4 expression was low.

## 4 Peptide-based CXCR4 radiopharmaceuticals

### 4.1 T140 and FC131 derivatives

**FIGURE 7:**
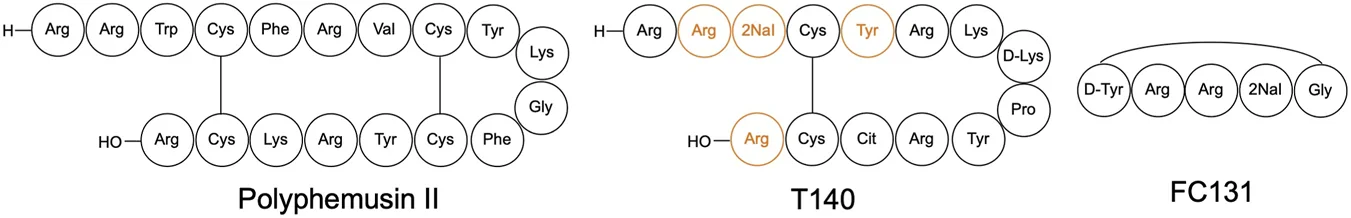
Sequence of polyphemusin II and its downsized analogues T140 and FC131. Required residues for CXCR4 inhibition of T140 are shown in orange.

Selected radiopharmaceuticals using peptide scaffolds derived from the Polyphemusin II peptide are summarized in Table 3. The peptides T140 and FC131 were discovered by downsizing the antimicrobial peptide Polyphemusin II through SAR studies (Figure 7) (Tamamura et al., 2008). T140 is a 14-mer peptide cyclized by a covalent disulfide bridge between two cysteine residues. FC131 is an end-to-end cyclic pentapeptide discovered from a conformationally constrained library approach using L- and D-amino acid substitutions of key CXCR4 binding residues, along with the incorporation of a glycine residue for rigidity (Fujii et al., 2003). The authors showed that the linear analogue had reduced activity which underscores the importance of a cyclic structure for this class of inhibitors. Early efforts in radiotracer design focused on T140 derivatives. AcTZ14011 was designed based on SAR studies and functionalized with a diethylenetriaminepentaacetic acid (DTPA) chelator for ^111^In labeling (Hanaoka et al., 2006). Although [^111^In]In-AcTZ14011 demonstrated nanomolar *in vitro* binding affinity to CXCR4, *in vivo* studies showed low tumor uptake and high accumulation in the liver, spleen and kidneys.

**TABLE 3:** Selected CXCR4 radiopharmaceuticals related to the T140 and FC131 cyclic peptides. The compound described is the lead compound selected for the study. Tumor-to-organ ratios for muscle (T/M), blood (T/B), kidneys (T/K) and liver (T/L). Uptake values are represented as % of injected activity per Gram of tissue (%IA/g) or mean standardized uptake values (SUV_mean_).

Compound	Scaffold and modifications	Binding properties^a^	Physicochemical properties	*In vivo* biodistribution^b^	References
Diagnostic					
[^111^In]In-DTPA-Ac-TZ14011	Polyphemusin II -DTPA chelator	ND	ND	AsPC-1 (PDAC) 60 min p.i: 0.51 ± 0.08 %IA/g (T)	(Hanaoka et al., 2006)
[^68^Ga]Ga-Pentixafor	CRPC4 -NH methylation -D-amino acids	^*125*^ *I-CPCR4 competition, IC*_*50*_: 5 ± 1 nM (Jurkat cells, 0.1 nM) ^*125*^ *I-FC131 competition, IC*_*50*_^*†*^: 24.8 ± 2.5 nM (Jurkat cells, 0.1 nM)	RCP: ND LogD_7.4_: −2.9 ± 0.08 MA: ND	OH1 (SCLC) 60 min p.i.: 6.16 ± 1.16 %IA/g (T), 5.7 (T/B), 2.9 (T/K), 3.3 (T/L)	(Demmer et al., 2011b; Gourni et al., 2011; Schottelius et al., 2017)
[^68^Ga]Ga-25	CRPC4 -Multimers	^*125*^ *I-CPCR4 competition, IC*_*50*_: 15 ± 3 nM (Jurkat cells, 0.1 nM)	ND	OH1 (SCLC) 60 min p.i.: 2.08 ± 0.48 %IA/g (T), 1.1 (T/B), 0.6 (T/K), 0.1 (T/L)	(Demmer et al., 2011a)
[^64^Cu]Cu-NOTA-Pentixather	iodoCRPC4 -Iodo-substituted D-Tyr^1^ -NOTA chelator	^*125*^ *I-FC131 competition, IC*_*50*_: 14.9 ± 2.1 nM (Jurkat cells, 0.1 nM)	RCP: >99% LogD_7.4_: −1.2 MA: 43 MBq/nmol	Daudi (Lymphoma) 90 min p.i.: 13.1 ± 1.5 %IA/g (T), 11.9 (T/B), 3.5 (T/K), 1.8 (T/L)	(Poschenrieder et al., 2017)
[^99m^Tc]Tc-PentixaTec	CRPC4 -N_4_ chelator -Dap-Arg-Ala-Abz linker	PentixaTec ^*125*^ *I-FC131 competition, IC*_*50*_: 0.6 ± 0.1 nM (Jurkat cells, 0.1 nM) ^*125*^ *I-CPCR4.3 competition, IC*_*50*_: 66.4 ± 16.3 nM (HEK:mCXCR4 cells, 0.1 nM) *[* ^*99m*^ *Tc]PentixaTec:* *FC131 competition*, *IC*_*50*_: 10.2 ± 2.4 nM (Jurkat cells, 0.1 nM)	RCP: >95% LogD_7.4_: −1.75 MA: ND	Jurkat (Lymphoma) 60 min p.i.: 8.6 ± 1.3 %IA/g (T), 5.4 (T/B), 0.3 (T/K), 1.1 (T/L)	(Konrad et al., 2023)
[^68^Ga]Ga-DOTA-r-a-ABA-CPCR4	CPCR4 -Arg-Ala-ABA linker	^*125*^ *I-FC131 competition*, *IC*_*50*_: 0.4 ± 0.1 nM (Jurkat cells, 0.1 nM)	RCP: ND LogD_7.4_: −3.6 ± 0.06 MA: ND	Daudi (Lymphoma) 60 min p.i.: 11.7 ± 1.3 %IA/g (T), 3.0 (T/B), 1.01 (T/K), 2.1 (T/L)	(Osl et al., 2020)
Therapeutic					
[^177^Lu]Lu-Pentixather	iodoCRPC4 -Iodo-substituted D-Tyr^1^	^*125*^ *I-FC131 competition*, *IC*_*50*_: 14.6 ± 1 nM (Jurkat cells, 0.1 nM) ^*125*^ *I-CPCR4.3 competition*, *IC*_*50*_^*†*^: >2000 nM (HEK:mCXCR4 cells, 0.1 nM)	RCP: >98% LogD_7.4_: −1.76 MA: ND	Daudi (Lymphoma) 60 min p.i.: 12.4 ± 3.7 %IA/g (T), 9.5 (T/B), 3.6 (T/K), 1.2 (T/L) 48 h p.i.: 3.27 ± 0.41 %IA/g (T), 164 (T/B), 2.7 (T/K), 0.4 (T/L)	(Schottelius et al., 2017; Konrad et al., 2023)
[^177^Lu]Lu-DOTA-r-a-ABA-CPCR4	CPCR4 -Arg-Ala-ABA linker	^*125*^ *I-FC131 competition, IC*_*50*_: 1.5 ± 0.1 nM (Jurkat cells, 0.1 nM)	RCP: ND LogD_7.4_: −3.0 ± 0.13 MA: ND	Daudi (Lymphoma) 60 min p.i.: 18.3 ± 3.7 %IA/g (T), 12.2 (T/B), 1.9 (T/K), 1.5 (T/L) 48 h p.i.: 8.81 ± 0.99 %IA/g(T), 881 (T/B), 2.9 (T/K), 1.0 (T/L)	(Osl et al., 2020)
[^161^Tb]Tb-Pentikra	iodoCPCR4 - D-Lys-D-Arg-Ala-ABA linker	^*68*^ *Ga-Pentixafor competition (Precursor), IC*_*50*_: 22.0 ± 6.66 nM (CHO:hCXCR4)	RCP: >95% LogD_7.4_: −3.06 ± 0.09 MA: ND	CHO:hCXCR4 (Engineered cell line) 6h p.i.: 23.63 ± 3.28 %IA/g (T), 125 (T/B), 1.1 (T/K), 0.8 (T/L) 48 h p.i.: 17.97 ± 2.17 %IA/g (T), 225 (T/B), 3.4 (T/K), 0.6 (T/L)	(Yu et al., 2026)

^a^ Values reported as half-maximal inhibitory concentration (IC50) are only directly comparable when determined under the same experimental conditions. The cell line and concentration of the labeled ligand are indicated when available.

^b^ Tumor uptake and tumor-to-organ ratios are reported for different tumor models that may exhibit varying levels of CXCR4 expression.

RCP, Radiochemical purity; T, Tumor uptake; PDAC, Pancreatic ductal adenocarcinoma; SCLC, Small cell lung carcinoma; ND, Not disclosed.

**FIGURE 8:**
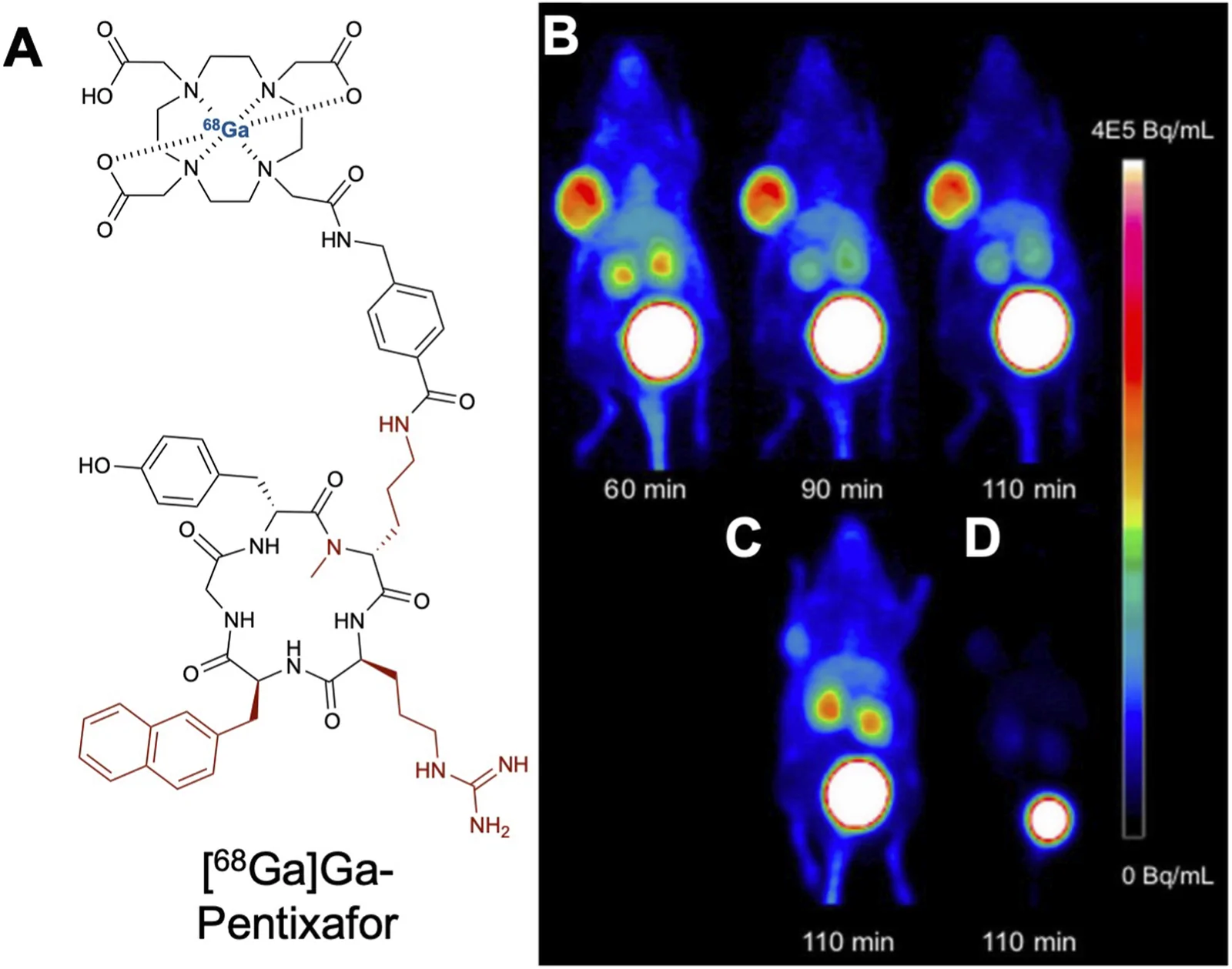
**(A)** Structure of [^68^Ga]Ga-Pentixafor. Chemical modifications and essential residues for binding are shown in red. **(B)** PET dynamic imaging (0–110 min) after the intravenous injection of [^68^Ga]Ga-Pentixafor (9.6 MBq) in OH1 tumor-bearing mice. **(C)** PET summation images (90–110 min) after co-injection of [^68^Ga]Ga-Pentixafor (13.7 MBq) and 50 µg of FC131 in OH1 tumor-bearing mice. **(D)** PET summation images (90–110 min) after co-injection of [^68^Ga]Ga-Pentixafor (7.4 MBq) and 50 µg of AMD3100 in OH1 tumor-bearing mice. Scale bar is shown as Bq/mL. Adapted with permission from (Gourni et al., 2011). Copyright 2011 Society of Nuclear Medicine and Molecular Imaging.

The FC131 scaffold has been extensively studied for CXCR4 radiotheranostics development. The ^68^Ga-labeled cyclic peptide (CPCR4-2, later named Pentixafor) was developed using the sequence c(-D-Tyr-D-[NMe]Orn-Arg-2-NaI-Gly-), a 4-aminomethylbenzoic acid (AMBA) linker and a DOTA chelator (Demmer et al., 2011b). Key modifications of the FC131 scaffold to improve binding affinity were methylation of the D-Tyr^1^-D-Orn^2^ peptide bond and substitution of a D-amino acid in position 2. Interestingly, the non-radioactive ^nat^Ga-complexed standard showed a >30-fold improved binding compared to the precursor (IC_50_ = 5 ± 1 nM vs. >100 nM), indicating that the chelator-metal complex significantly impacts receptor binding. [^68^Ga]Ga-Pentixafor showed high tumor uptake (6.16 ± 1.16 %IA/g) leading to high tumor/liver ratios (3.31 ± 0.32), tumor/kidneys (0.32 ± 0.10) and tumor/blood (5.81 ± 0.88) at 1 h p.i in a human small lung carcinoma xenograft model (Gourni et al., 2011) (Figure 8). However, fast tumor clearance and poor binding affinity with other radioisotopes have limited its translation as a therapeutic compound.

[^68^Ga]Ga-Pentixafor was initially designed to include a DOTA chelator, which may contribute to CXCR4 binding through its structural similarity to cyclams while providing improved hydrophilicity compared to other radiolabeling strategies (Demmer et al., 2011b; Schottelius et al., 2019). [^68^Ga]Ga-Pentixafor has been extensively investigated as a molecular imaging drug for diagnosis and RPT treatment planning in patients with hematological malignancies such as multiple myeloma, lymphomas and leukemias (Lapa et al., 2017b; Luo et al., 2019; Mayerhoefer et al., 2021; Buck et al., 2022). Also, [^68^Ga]Ga-Pentixafor showed high and specific uptake in primary aldosteronism nodules and adrenocortical carcinoma lesions (Bluemel et al., 2017; Buck et al., 2022; Gao et al., 2023). Among non-oncological applications, CXCR4 PET imaging is currently under investigation for identification of atherosclerotic plaques and its utility as a biomarker for risk stratification among patients with acute myocardial infraction (Hyafil et al., 2017; Kircher et al., 2020; Diekmann et al., 2025). Subsequent Pentixafor-based derivatives focused on modifying linkers and chelators to generate thermodynamically stable complexes with other radioisotopes. ^99m^Tc-labeled derivatives were designed for scintigraphy/SPECT imaging as an alternative approach to PET imaging. Systematic evaluation of three different chelators (mercaptoacetyl-seryl-seryl-serine, hydrazinonicotinamide or N_4_) combined with arginine-based peptide linkers led to the development of [^99m^Tc]Tc-PentixaTec (Konrad et al., 2023). [^99m^Tc]Tc-PentixaTec showed high *in vivo* tumor uptake in a lymphoma model but also exhibited high uptake values in liver (7.7 ± 0.7 %IA/g), kidneys (37.4 ± 2.7 %IA/g), spleen (5.6 ± 0.8 %IA/g) and lungs (4.0 ± 0.6 %IA/g). In a first-in-human study, it resulted in high-contrast SPECT imaging and dosimetry results showed that the spleen received the highest dose followed by kidneys and bone marrow. [^99m^Tc]Tc-PentixaTec is currently under evaluation for cardiovascular and oncology application (Enke et al., 2024; Liebich et al., 2024).

Different chelator-metal complexes significantly impacted CXCR4 binding affinity of (Poschenrieder et al., 2016). While DOTA and 1,4,7-triazacyclononane-1,4,7-triacetic acid (NOTA) complexes with Ga^3+^ and Bi^3+^ preserved IC_50_ values, complexes with Lu^3+^, Y^3+^, AlF^2+^and Cu^2+^or other chelators had poorer binding affinity. The Pentixafor analogue, Pentixather, contains an iodine substitution at the D-Tyr^1^ residue and showed good binding affinity values in complex with Lu^3+^, Y^3+^ and Bi^3+^. Pentixather was reported as an optimized scaffold for the development of therapeutic radiopharmaceuticals allowing further chemical modifications (Schottelius et al., 2017). Pentixather exhibited similar IC_50_ values when conjugated to NOTA-derived chelators suitable for ^64^Cu labeling (Poschenrieder et al., 2017). [^64^Cu]Cu-NOTA-Pentixather resulted in high-contrast PET images at 1 h p.i. However, the development of a matched ^64^Cu/^67^Cu theranostic pair was limited by rapid tumor clearance.

**FIGURE 9:**
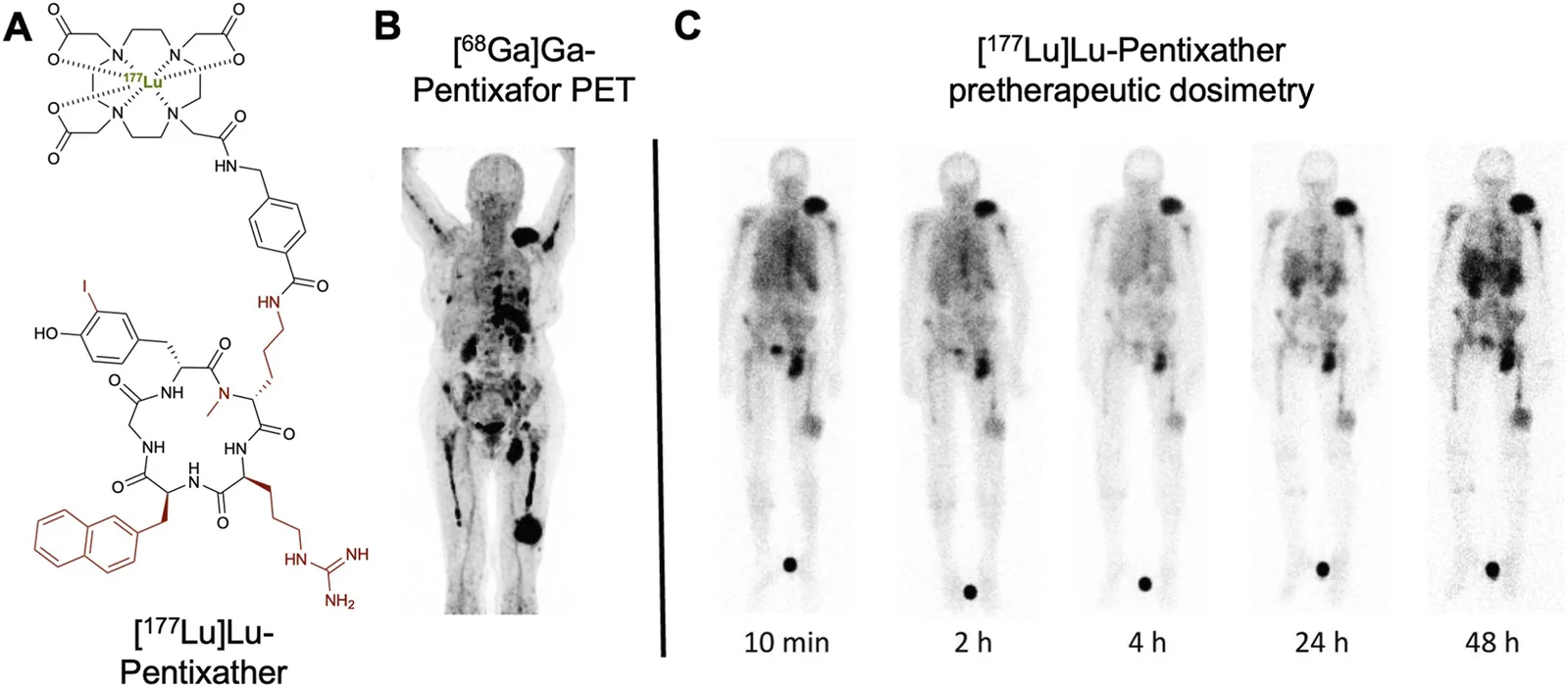
**(A)** Structure of [^177^Lu]Lu-Pentixather. Chemical modifications and essential residues for binding are shown in red. **(B)** [^68^Ga]Ga-Pentixafor PET/CT images of a patient with relapsed refractory multiple myeloma. **(C)** [^177^Lu]Lu-Pentixather scintigraphic images of same patient at different timepoints of pretherapeutic dosimetry. Adapted with permission from (Lapa et al., 2017a) in accordance to the Creative Commons Attribution (CC BY-NC) License. Copyright 2017 Ivyspring International Publisher.

The therapeutic radiopharmaceutical, [^177^Lu]Lu-Pentixather, resulted in sustained tumor retention up to day 7 post-injection in a lymphoma xenograft model (Schottelius et al., 2017). Liver uptake was higher at 1 h compared to previously reported values for [^68^Ga]Ga-Pentixafor (10.3 ± 0.8 %IA/g vs. 1.85 ± 0.24 %IA/g). In the same study, pre-therapeutic dosimetry data from a patient with multiple myeloma showed substantially higher absorbed doses in kidneys, spleen and bone marrow compared to dosimetry estimates extrapolated from mouse data. This highlights limitations of mouse models to predict human dosimetry. Imaging and biodistribution studies across different xenograft models with Pentixafor and Pentixather showed low spleen and bone marrow uptake, likely due to the poor cross reactivity between human and murine CXCR4 (Wester et al., 2015). In contrast, imaging of [^177^Lu]Lu-Pentixather in patients with CXCR4-expressing cancers showed high bone marrow uptake, reflecting physiological CXCR4 expression in hematopoietic tissues and contributing to myeloablation at therapeutic doses (Figure 9) (Lapa et al., 2017a; 2019; Schottelius et al., 2017; Maurer et al., 2019; Hänscheid et al., 2021; Buck et al., 2023). CXCR4 RPT has been investigated in Germany in a myeloablative setting followed by hematopoietic stem cell transplant, preferentially with the shorter-lived radioisotope ^90^Y to reduce the duration of radiation exposure in the bone marrow prior to stem cell engraftment. Recently, CXCR4 RPT has been evaluated as part of conditioning regime prior stem cell transplant in patients with acute myeloid leukemia (Braitsch et al., 2025).

The Pentixather scaffold continues to be used in preclinical research for the development of novel theranostic pairs. [^68^Ga]Ga/[^161^Tb]Tb-Pentikra was designed to contain a D-Lys-D-Arg-Ala-ABA linker coupled to a DOTA chelator (Yu et al., 2026). In a head-to-head comparison, [^68^Ga]Ga-Pentikra resulted in 3-fold higher tumor uptake compared to [^68^Ga]Ga-Pentixather at 1 h p.i. However, higher uptake was observed in the kidneys and liver. The theranostic pair [^203/212^Pb]Pb-Pentixather was recently evaluated in a human CD34^+^ bone marrow engrafted preclinical model with small cell lung carcinoma xenografts. This study showed effective tumor growth control with alpha-RPT but resulted in reduction of peripheral blood cell counts and human CD45^+^ cells in bone marrow aspirates after 2 weeks post-treatment (Christensen et al., 2025).

Linker modifications and dimerization strategies were also used to improve receptor binding affinity and tumor retention. *In vitro* binding affinity was modulated by the linker length (Demmer et al., 2011a). The lead candidate [^68^Ga]Ga-25 was evaluated *in vivo* but exhibited unfavorable tumor-to-organ ratios. Molecular docking studies using the published CXCR4 crystal structure (PDB: 3OE0) showed a 2-site binding model, with one monomer occupying the CRS2 major pocket with the Orn^2^ residue interacting with D187 with the second monomer extending towards the extracellular surface. Modifications of the AMBA linker for a 4-aminobenzoic acid (ABA) linker in both the Pentixafor and Pentixather compounds resulted in improved binding affinity in complex with Lu^3+^ (Osl et al., 2020). Moreover, the authors found that an anionic-charged linker improved binding affinity compared to a cationic-charged linker. Although these compounds exhibited similar tumor uptake compared to [^68^Ga]Ga-Pentixafor, kidneys and liver accumulation were higher. [^177^Lu]Lu-DOTA-r-a-ABA-CPCR4 and [^177^Lu]Lu-DOTA-r-a-ABA-iodoCPCR4 had slower tumor clearance at 48 h p.i. compared to [^177^Lu]Lu-Pentixafor.

### 4.2 LY2510924-derived peptides

**FIGURE 10:**
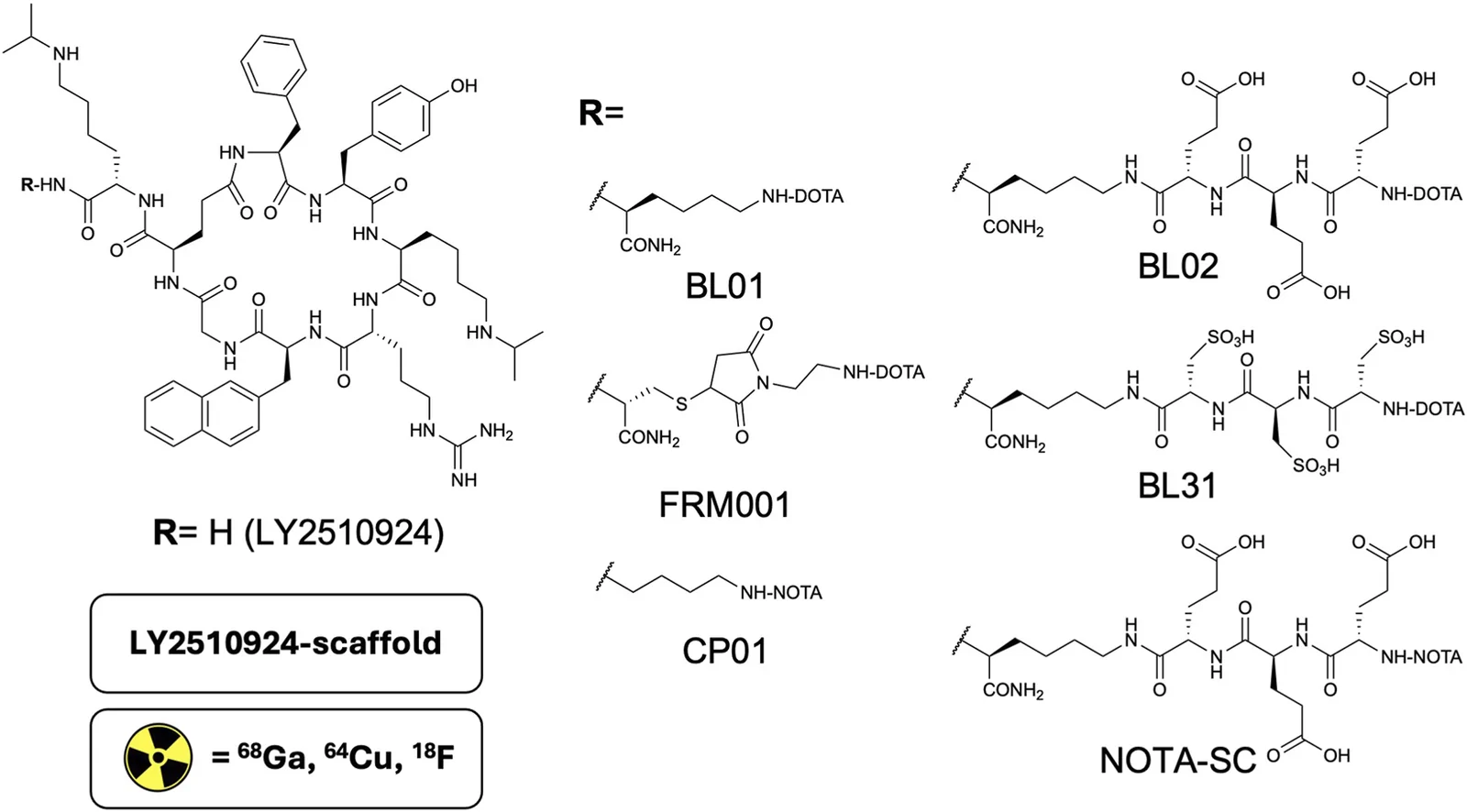
Selected structures of LY2510924-derived radiopharmaceuticals labeled with ^64^Cu, ^68^Ga and ^18^F.

The cyclic 8-mer peptide LY2510924 (c[Phe-Tyr-Lys(iPr)-D-Arg-2-NaI-Gly-D-Glu]-Lys(iPr)-NH_2_) is a potent CXCR4 inhibitor synthesized through rational design (Peng et al., 2015). LY2510924 demonstrated potent binding inhibition of SDF-1α and ERK/Akt phosphorylation. Peng et al. proposed 7 potential ligand-receptor interactions with residues from both the minor and major pocket. Selected radiopharmaceuticals using the LY2510924 scaffold are summarized in Table 4 and Figure 10.

**TABLE 4:** Selected CXCR4 radiopharmaceuticals derived from the LY2510924 cyclic peptide. The compound described is the lead compound selected for the study. Tumor-to-organ ratios for muscle (T/M), blood (T/B), kidneys (T/K) and liver (T/L). Uptake values are represented as % of injected activity per Gram of tissue (%IA/g) or mean standardized uptake values (SUV_mean_).

Compound	Scaffold and modifications	Binding properties^a^	Physicochemical properties	*In vivo* biodistribution^b^	References
Diagnostic					
[^68^Ga]Ga-BL01	LY2510924 -C-terminus Lys linker -DOTA chelator	^*125*^ *I-SDF-1α competition, IC*_*50*_ *:* 21.2 ± 15.9 nM (CHO:hCXCR4, 0.01 nM)	RCP: >99% LogD_7.4_: −3.36 ± 0.09 MA: 40 ± 11 MBq/nmol	Daudi (Lymphoma) 60 min p.i.: 10.20 ± 2.56 %IA/g (T), 4.0 (T/B), 2.0 (T/K), 12.8 (T/L)	(Lau et al., 2019)
[^68^Ga]Ga-FRM001	LY2510924 -maleido-mono-amide-DOTA chelator	^*125*^ *I-SDF-1α competition, IC*_*50*_ *:* 2.26 ± 0.51 nM (CCRF-CEM, 0.25 nM)	RCP: >95% LogD_7.4_: −3.23 ± 0.05 MA: 19–20 MBq/nmol	CCRF-CEM (Lymphoblastic leukemia) 60 min p.i.: 12.02 ± 1.99 %IA/g (T), 58 (T/B), 2.5 (T/K), 0.8 (T/L)	(Suzuki et al., 2019)
[^18^F]F-BL08	BL02 -AMBF_3_	^*125*^ *I-SDF-1α competition, IC*_*50*_ *:* 11.6 ± 7.0 nM (CHO:hCXCR4 cells, 0.01 nM)	RCP: >97% LogD_7.4_: −3.45 ± 0.33 MA: 94.17 ± 25.15 MBq/nmol	Daudi (Lymphoma) 60 min p.i.: 7.60 ± 1.38 %IA/g (T), 21.5 (T/B), 2.2 (T/K), 13.05 (T/L)	(Kwon et al., 2021)
[^64^Cu]Cu-NOTA-CP01	BL01 -NOTA chelator	*Homologous competition, IC*_*50*_ *:* 1.61 ± 0.96 nM (EC109 cells) *Saturation assay, K*_*D*_ *:0.27* ± 0.14 nM (EC109 cells)	RCP: >99% LogD_7.4_: −3.44 ± 0.12 MA: 10.5–21 MBq/nmol	EC109 (ESCC) 24 h p.i.: 2.97 ± 1.01 %IA/g (T), 7.4 (T/B), 1.0 (T/K), 0.6 (T/L)	(Peng et al., 2021)
[^68^Ga]Ga-BL02	BL01 -Tri-Glu linker	^*125*^ *I-SDF-1α displacement, IC*_*50*_: 27.9 ± 12.5 nM (CHO:hCXCR4, 0.01 nM)	RCP: >99% LogD_7.4_: −4.20 ± 0.44 MA: 258.06 ± 89.63 MBq/nmol	Daudi (Lymphoma) 60 min p.i.: 9.14 ± 1.49 %IA/g (T), 34.8 (T/B), 2.4 (T/K), 16.2 (T/L) Z138 (Lymphoma) 60 min p.i.: 12.94 ± 1.28 %IA/g (T), 43.1 (T/B), 3.7 (T/K), 26.9 (T/L)	(Kwon et al., 2022a)
[^18^F]AlF-NOTA-SC	BL02 -NOTA chelator	*SDF-1α* ^*AF647*^ *competition, IC*_*50*_ *:* 14.38 ± 2.65 nM (U87 cells, 100 ng/mL)	RCP: >98% LogD_7.4_: ND MA: 16.35 ± 3.61 MBq/nmol	MM.1S (Multiple myeloma) 50–60 min p.i.: 1.95 ± 0.11 tumor SUV_mean_ MM.1S, orthotopic 50–60 min p.i.: 2.12 ± 1.24 SUV_mean_	(Spahn et al., 2025)
[^68^Ga]Ga-BL34	LY2510924 -D-Ala^2^, Lys(Cysteic acid-DOTA)^7^	^*125*^ *I-SDF-1α competition, IC*_*50*_: 10.3 ± 5.7 nM (CHO:hCXCR4, 0.01 nM)	RCP: >95% LogD_7.4_: ND MA: 346 ± 206 MBq/nmol	Z138 (Lymphoma) 60 min p.i.: 15.09 ± 3.13 %IA/g (T), 56.0 (T/B), 6.4 (T/K), 17.9 (T/L)	(Kwon et al., 2026)
[^68^Ga]Ga-JH120061	BL34 -4-Pal^7^ -Gly^2^	*Pentixafor* ^*FITC*^ *competition, IC*_*50*_ *:* 3.02 ± 0.12 nM (HT-1080:hCXCR4, 10 nM Pentixafor)	RCP: >95% LogD_7.4_: ND MA: ND	Z138 (Lymphoma) 60 min p.i.: 31.3 ± 9.12 %IA/g (T), 58.0 (T/B), 9.34 (T/K), 22.2 (T/L)	(Wang et al., 2026)
Therapeutic					
[^177^Lu]Lu-BL01	LY2510924 -C-terminal Lys-linker -DOTA chelator	^*125*^ *I-SDF1a competition, IC*_*50*_ *:* 7.1 ± 1.7 nM (CHO:hCXCR4, 0.01 nM)	RCP: >99% LogD_7.4_: −3.34 ± 1.1 MA: 120 ± 21 MBq/nmol	Daudi (Lymphoma) 60 min p.i.: 14.0 ± 1.12 %IA/g (T), 6.14 (T/B), 2.30 (T/K), 1.37 (T/L) 72 h p.i.: 3.62 ± 0.68 %IA/g (T), 127 (T/B), 2.99 (T/K), 0.74 (T/L)	(Lau et al., 2019)
[^177^Lu]Lu-BL02	BL01 -Tri-Glu linker	^*125*^ *I-SDF-1α competition, IC*_*50*_: 22.7 ± 8.4 nM (CHO:hCXCR4, 0.01 nM)	RCP: >99% LogD_7.4_: ND MA: 247.65 ± 28.95 MBq/nmol	Z138 (Lymphoma) 60 min p.i.: 17.2 ± 3.04 %IA/g (T), 39.0 (T/B), 4.5 (T/K), 18.5 (T/L) 72 h p.i.: 3.57 ± 0. 56 %IA/g (T), 613 (T/B), 6.38 (T/K), 8.32 (T/L)	(Kwon et al., 2022a)
[^177^Lu]Lu-BL34	LY2510924 -D-Ala^2^, Lys(Cysteic acid-DOTA)^7^	^*125*^ *I-SDF1a competition, IC*_*50*_: 24.3 ± 11 nM (CHO:hCXCR4, 0.01 nM)	RCP: >95% LogD_7.4_: ND MA: 249 ± 65 MBq/nmol	Z138 (Lymphoma) 60 min p.i.: 14.3 ± 2.51 %IA/g (T), 33.1 (T/B), 5.28 (T/K), 20.5 (T/L) 24 h p.i.: 9.39 ± 1.80 %IA/g (T), >900 (T/B), 10.7 (T/K), 17.7 (T/L) 72 h p.i.: 4.00 ± 1.26 %IA/g (T), 582 (T/B), 8.61 (T/K), 10.4 (T/L)	(Kwon et al., 2026)
[^177^Lu]Lu-JH120061	BL34 -4-Pal^7^ -Gly^2^	*Pentixafor* ^*FITC*^ *competition, IC*_*50*_ *:* 3.93 ± 0.08 nM (HT1080, 10 nM Pentixafor)	RCP: >95% LogD_7.4_: ND MA: ND	Z138 (Lymphoma) 60 min p.i.: 15.9 ± 3.30 %IA/g (T), 54.9 (T/B), 3.84 (T/K), 12.2 (T/L) 24 h p.i.: 9.66 ± 3.15 %IA/g (T), >1000 (T/B), 4.20 (T/K), 11.5 (T/L)	(Wang et al., 2026)

^a^ Values reported as half-maximal inhibitory concentration (IC50) are only directly comparable when determined under the same experimental conditions. The cell line and concentration of the labeled ligand are indicated when available.

^b^ Tumor uptake and tumor-to-organ ratios are reported for different tumor models that may exhibit varying levels of CXCR4 expression.

RCP, Radiochemical purity; T, Tumor uptake; ESCC, Esophageal squamous cell carcinoma.

**FIGURE 11:**
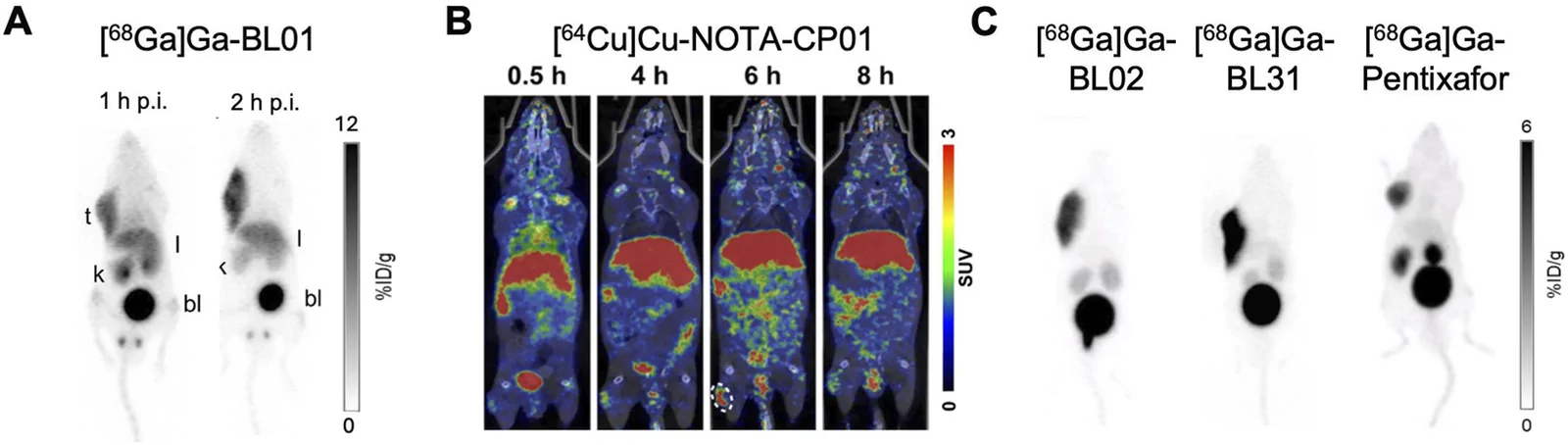
**(A)** PET images of [^68^Ga]Ga-BL01 at 1 h and 2 h p.i. in mice engrafted with Z138 tumors (left shoulder). Images are shown as maximum intensity projection and scale bar is shown as %ID/g. Adapted with permission from (Lau et al., 2019). Copyright 2019 American Chemical Society. **(B)** Time-course PET images of [^64^Cu]Cu-NOTA-CP01 at 0.5, 4, 6 and 8 h p.i. in mice engrafted with EC109 tumors (left flank, dashed circle). Scale bar is shown as SUV. Adapted with permission from (Peng et al., 2021). Copyright 2021 American Chemical Society. **(C)** PET images of ^68^Ga-labeled BL02, BL31 and Pentixafor at 1 h p.i. in mice engrafted with Z138 tumors (left shoulder). Image are shown as maximum intensity projection and scale bar is shown as %ID/g. Adapted with permission from (Kwon et al., 2022c) in accordance to the Creative Commons Attribution License (CC BY).

Our group reported the synthesis of a radiopharmaceutical in which DOTA was coupled to the C-terminus of LY2510924 through a lysine residue (BL01) (Lau et al., 2019). Complexation with Ga^3+^ or Lu^3+^ did not significantly affect CXCR4 binding affinity, and both ^68^Ga and ^177^Lu-labeled BL01 showed similar biodistribution profiles. Despite [^68^Ga]Ga-BL01 exhibited high uptake in tumor (10.2 ± 2.56 %IA/g), it show high uptake in normal organs such as lungs, spleen and liver (Figure 11A). Suzuki et al. concurrently reported a maleimido-mono-amide DOTA-conjugated to the C-terminus of LY2510924 using a cysteine linker (FRM001) (Suzuki et al., 2019). Their data revealed that precursor and non-radioactive standards (Ga^3+^, Lu^3+^ and Y^3+^) exhibited similar binding affinities (IC_50_ = 0.89–2.26 nM) compared to LY2510924 and consistently lower values compared to AMD3100, AMD3465, FC131 and BL-8040 in a competition assay performed using the identical experimental conditions. [^67^Ga]Ga-FRM001 exhibited the highest uptake in the tumor (12.0 ± 1.99 %IA/g) and liver (16.1 ± 2.66) at 1 h after injection in a human leukemic lymphoblast xenograft model. ^68^Ga-labeled FRM001 resulted in high contrast PET images validating the biodistribution results. These initial studies highlighted the potential of LY2510924 as a versatile scaffold for the development of CXCR4-targeted theranostic pairs.

Further modifications of the LY2510924 scaffold include different radioisotopes, chelators and linkers. [^64^Cu]Cu-CP01 was coupled to a NOTA chelator for copper radiolabeling *via* a butane-1,4-diamine linker. While good binding affinity was reported using a membrane protein assay, PET images showed poor tumor-to-organ ratios (Figure 11B) (Peng et al., 2021). Systematic linker modifications of BL01 showed that anionic residues such as a tri-glutamate (BL02), tri-homoglutamate (BL17), tri-D-glutamate (BL20), tri-aspartate (BL25) and tri-cysteic acid (BL31) resulted in improved tumor-to-organ ratios compared to a piperidine-based cationic linker (BL06) (Kwon et al., 2022c). [^68^Ga]Ga-BL02 and [^68^Ga]Ga-BL31 PET images showed lower accumulation in normal organs compared to [^68^Ga]Ga-BL01. Compared to [^68^Ga]Ga-Pentixafor, these tracers showed similar tumor uptake and lower kidney uptake in a head-to-head study (Figure 11C). ^18^F-labeled CXCR4 compounds derived from the BL02 scaffold using a carboxy-ammoniomethyl-trifluoroborate (BL08) or an alkyne ammoniomethyl-trifluoroborate (AMBF_3_, BL09) prosthetic group showed high contrast PET images at 1 h p.i. with the former showing increased hydrophilicity and reduced kidney uptake (Kwon et al., 2021). Spahn et al. developed [^18^F]AlF-NOTA-SC and showed clear delineation of CXCR4-expressing tumor cells in both a subcutaneous model and a systemic model at 50–60 min after injection (Spahn et al., 2025).

**FIGURE 12:**
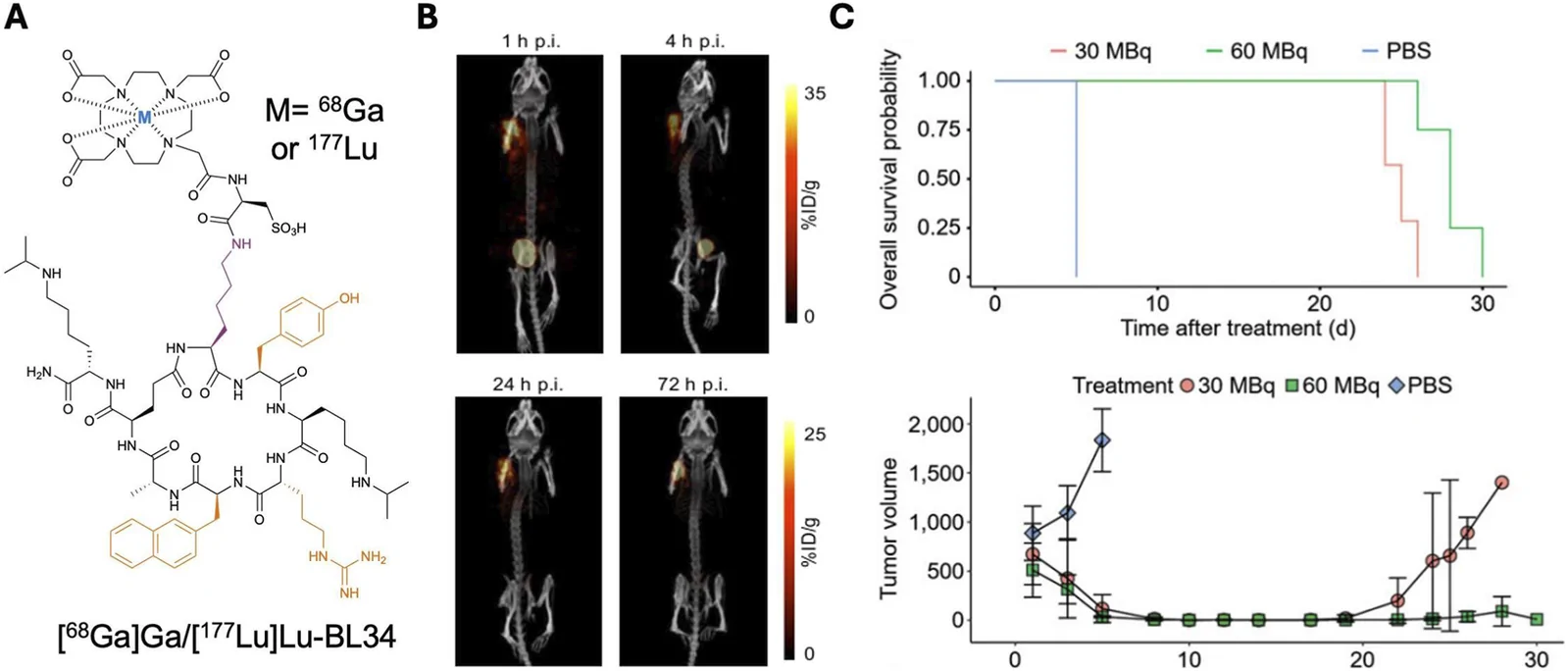
**(A)** Structure of the theranostic pair [^68^Ga]Ga/[^177^Lu]Lu-BL34. Essential residues for binding are shown in orange and modified residues are shown in purple. **(B)** SPECT images of [^177^Lu]Lu-BL34 at 1, 4, 24 and 72 h p.i. Scale bar is shown as %ID/g. **(C)** Kaplan-Meier survival curves and tumor growth curves of mice injected with 30 or 60 MBq of [^177^Lu]Lu-BL34 and PBS (control group). Tumor volume is shown as mean ± SD. Adapted with permission from (Kwon et al., 2026) in accordance to the Creative Commons Attribution License (CC BY). Copyright 2026 Society of Nuclear Medicine and Molecular Imaging.

The therapeutic efficacy of [^177^Lu]Lu-BL02 was evaluated in a mantle cell lymphoma model (Z138 tumor model) resulting in significant tumor growth regression followed by tumor regrowth (Kwon et al., 2022a). A series of optimized CXCR4 radiopharmaceuticals derived from BL31 were synthesized aiming to further improve binding affinity and improve their biodistribution. Detailed structure activity optimization studies showed good binding when positioning the cysteic-acid linker in position 7 by replacing the Phe^7^ for a Lys^7^ and substituting Gly^2^ for D-Ala^2^ (BL34, IC_50_: 24.2 ± 3.5 nM) (Kwon et al., 2026). [^177^Lu]Lu-BL34 showed sustained tumor uptake at 1, 4, 24 and 72 h p.i. by SPECT imaging and potent tumor growth inhibition and improved overall survival in mice (Figure 12). However, [^177^Lu]Lu-BL34 failed to achieve sustained tumor control. Substitution of the DOTA chelator in BL34 with a carboxypropyl-AMBF_3_ (BL40) for facile ^18^F-labeling resulted in high RCP (>97%) and high tumor uptake (21.1 ± 2.1 %IA/g) in a Z138 xenograft model (Kwon et al., 2022b). [^18^F]F-BL40 is currently being evaluated in a Phase1/2 study (NCT06224309) for its utility to identify CXCR4-expressing tumors and safety profile. Also, substituting D-Ala^2^ for Gly^2^ and Lys^7^ for 4-Pal2^7^ for linker attachment in the BL34 scaffold (JH120061) resulted in good binding properties (IC_50_: 6.0 ± 0.4 nM) and high specificity for CXCR4 (Wang et al., 2026). ^68^Ga-labeled JH120061 showed 2-fold high tumor uptake in a Z138 tumor model (31.3 ± 9.12 %IA/g) compared to the ^177^Lu labeled counterpart (15.9 ± 3.30 %IA/g).

Overall, the LY2510924 scaffold is suitable for diverse chemical modifications, making it a promising candidate for developing radiotheranostics. While LY2510924-derivatived radiopharmaceuticals have demonstrated positive preclinical results, their clinical performance requires further investigation.

### 4.3 Other nature-inspired peptides: CXCL12 and vMIP-II derivatives

**FIGURE 13:**
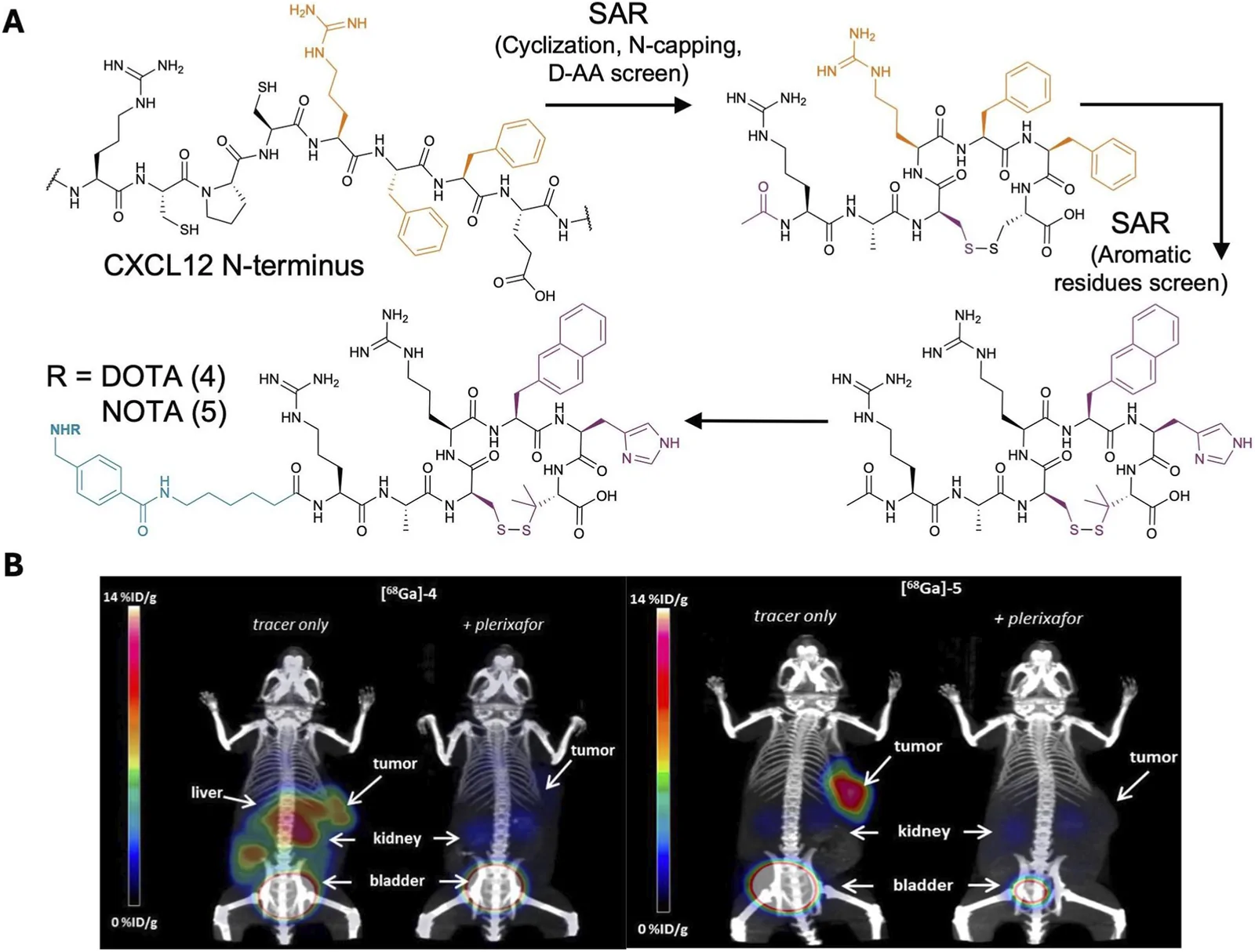
**(A)** Example structures of CXCR4 inhibitors and derived radiopharmaceuticals. Essential residues for binding are shown in orange, modified residues are shown in purple and linker is shown in blue. **(B)** PET images of [^68^Ga]Ga-4 and [^68^Ga]Ga-5 at 1 h p.i. in mice engrafted with Daudi tumors. Blocking was performed by co-injection of 50 µg of AMD3100. Image are shown as maximum intensity projection and scale bar is shown as %ID/g. Adapted with permission from (Trotta et al., 2021). Copyright 2021 American Chemical Society.

The structures of different natural CXCR4 ligands such as CXCL12 and the human herpesvirus 8-encoded chemokine vMIP-II have been investigated as scaffolds for CXCR4 at the imaging stage (Table 5). Radiopharmaceuticals derived from endogenous chemokine sequences require structural ridification, peptide bond stabilization and N-terminal capping to ensure *in vivo* stability and a suitable plasma half-life for imaging or therapy. The heptapeptide AcArg-Ala-c[D-Cys-Arg-2NaI-His-Pen] was developed through extensive SAR optimization and recently translated into a PET imaging agent (Figure 13A). The initial library of 19 cyclic peptides was designed around two cysteines for cyclization and a three-residue motif from the CXCL12 N-terminus. The lead candidate (RACRFFC, Arg-Ala-c[Cys-Arg-Phe-Phe-Cys]) resulted in selective CXCR4 binding but poor potency (IC_50_: 6.2 ± 1.3 µM) and stability (Portella et al., 2013). N-terminal acetylation improved plasma stability and binding and a D-amino acid scan identified D-Cys^3^ as a key substitution to improved binding (IC_50_: 53 ± 4 nM) (Di Maro et al., 2016). Additionally, substitution of the Cys^3^, Phe^5^, Phe^6^ and Cys^7^ residues (D-Cys^3^, 2NaI^5^, His^6^, Pen^7^[AcRACRFFC]) resulted in the latest iteration with good binding (IC_50_: 1.5 ± 0.5 nM) (Di Maro et al., 2017). This was chosen as a scaffold for the development of the first radiopharmaceuticals of this class. A Lys-AMBA linker was coupled to the N-terminus for conjugation of a DOTA (compound 4) or NOTA (compound 5) chelator (Trotta et al., 2021). These modifications reduced binding compared to the parental peptide; however complexation with Ga^3+^ partially restored binding (IC_50_: 49 ± 15 and 16 ± 4.2 nM). This is a recurring observation across different classes of CXCR4-targeted radiopharmaceuticals. Despite their antagonistic activity, the ^68^Ga-labeled compounds 4 and 5 showed 70% and 58% internalization after 90 min, respectively. [^68^Ga]Ga-4 and [^68^Ga]Ga-5 exhibited the highest uptake in tumor (5.61 ± 0.43 and 7.88 ± 1.43 %IA/g) and kidneys (3.27 ± 0.24 and 3.55 ± 0.42 %IA/g) at 1 h after injection (Figure 13B). [^18^F]AlF-labeling of NOTA-QHY-04 resulted in high-contrast PET images in several CXCR4-expressing preclinical tumor models (Cheng et al., 2024). In small patient cohorts, [^18^F]AlF-NOTA-QHY-04 PET images demonstrated rapid clearance and favorable tumor-to-background contrast across different malignancies. The highest uptake was observed in patients with diffuse large B cell lymphoma and small cell lung carcinoma, supporting further investigation of this scaffold in larger controlled clinical studies.

**TABLE 5:** Selected CXCR4 radiopharmaceuticals derived from CXCL12 and vMIP-II scaffolds. The compound described is the lead compound selected for the study. Tumor-to-organ ratios for blood (T/B), kidneys (T/K) and liver (T/L). Uptake values are represented as % of injected activity per Gram of tissue (%IA/g) or mean standardized uptake values (SUV_mean_).

Compound	Scaffold and modifications	Binding properties^a^	Physicochemical properties	*In vivo* biodistribution^b^	References
CXCL12 derivatives					
[^68^Ga]Ga-5	CXCL12 N-terminus -Arg-Ala-c[D-Cys-Arg-2NaI-His-Pen] -N-terminus HLys-AMBA-NOTA	^*125*^ *I-FC131 competition*, *IC*_*50*_: 15.6 ± 4.2 nM (Jurkat cells, 0.1 nM)	RCP: >98% LogD_7.4_: −1.6 MA: 38–45 MBq/nmol	Daudi (Lymphoma) 60 min p.i.: 7.88 ± 1.43 (T), 19.7 (T/B), 2.2 (T/K), 21.9 (T/L)	(Trotta et al., 2021)
[^18^F]AlF-NOTA-QHY-04	CXCL12 N-terminus (Compound 5) -Arg-Ala-c[D-Cys-Arg-2NaI-His-Pen] -N-terminus HLys-AMBA-NOTA	*Saturation assay, K*_*D*_ *:* 41.3 ± 5.5 nM (A549:hCXCR4 cells)	RCP: >98% LogD_7.4_: −2.21 ± 0.06 MA: 30 ± 3 MBq/nmol	Daudi (Lymphoma) 60 min p.i: ∼7.5 %IA/g tumor uptake^*^ H69 (colorectal carcinoma) 60 min p.i: ∼5 %IA/g tumor uptake^*^	(Cheng et al., 2024)
vMIP-II derivatives					
[^18^F]AlF-NOTA-DV1-k-(DV3)	DV1-k-DV3 -NOTA chelator	*SDF-1α* ^*AF647*^ *competition, IC*_*50*_ *:* 5.3 ± 0.9 nM (Jurkat cells, *25 ng/mL*) 33.4 ± 13.5 nM (U87:mCXCR4 cells)	RCP: >98% LogD: ND MA: 11.5 ± 2.5 MBq/nmol	U87.hCXCR4 (Engineered cell line) 75 min p.i.: 0.6 ± 0.2 SUV_mean_ (T), 4.6 (T/B), 0.02 (T/K), 0.1 (T/L)	(Luyten et al., 2021)
^18^F]AlF-NOTA-2xDV1(c11sc12s)	DV1-k- DV1 -S^11^, S^12^ -NOTA chelator	*SDF-1α* ^*AF647*^ *competition, IC*_*50*_ *:* 1.3 ± 0.2 nM (Jurkat cells, *25 ng/mL*), 7.2 ± 1.5 nM (U87:mCXCR4 cells, *25 ng/mL*)	RCP: >95% LogD: ND MA: 19.6 ± 3.5 MBq/nmol	U87.hCXCR4 (Engineered cell line) 75 min p.i.: 0.47 ± 0.09 SUV_mean_ (T), 9.4 (T/B), 0.03 (T/K), 0.04 (T/L)	(Spahn et al., 2024)

^a^ Values reported as half-maximal inhibitory concentration (IC50) are only directly comparable when determined under the same experimental conditions. The cell line and concentration of the labeled ligand are indicated when available.

^b^ Tumor uptake and tumor-to-organ ratios are reported for different tumor models that may exhibit varying levels of CXCR4 expression.

RCP, Radiochemical purity; T, Tumor uptake; (^*^) No raw values were reported in publication.

The chemokine vMIP-II was found to inhibit CXCR4 and other chemokine receptors. Although its sequence differs significantly from CXCL12, it adopts a similar mode of binding *via* the N-terminus to the CRS2 minor subpocket (Qin et al., 2015). Two N-terminal peptides, V1 (aa 1–21) and V3 (aa 1–10) contain key residues for CXCR4 inhibition (Zhou et al., 2000). In competition assays, the D-amino acid analogue (DV1) showed improved binding (IC_50_: 13 nM) compared to its L-form with further improvement upon dimerization of the fragments DV1 and (DV1-k-(DV3), IC_50_: 4 nM) (Zhou et al., 2002; Xu et al., 2013). The peptide D1-k(DV3) was used as a scaffold for the initial development of vMIP-II derived radiopharmaceuticals for which a DOTA/NOTA chelator was coupled to the ε-amino group of the C-terminal DV3 Lys (Luyten et al., 2021). Complexation with different non-radioactive isotopes (DOTA: Ga^3+^, Lu^3+^, Bi^3+^, La^3+^ or NOTA: AlF^2+^) showed little impact on their binding towards CXCR4, supporting their radiotheranostic application. Despite the higher *in vitro* binding affinity of AlF-NOTA-DV1-k-(DV3) compared to Ga-Pentixafor, AlF-NOTA-DV1-k-(DV3) showed 5-fold lower tumor uptake and significantly higher uptake in murine CXCR4 expressing organs such as liver, spleen and bone marrow. A second-generation radiopharmaceutical, [^18^F]AlF-NOTA-2×(DV1c11sc12s), was designed using the high-affinity DV1 monomers with a S^11^ and S^12^ substitutions to prevent oxidation within the tumor microenvironment and improve tumor retention (Spahn et al., 2024). However, no improvement in the *in vivo* profile was observed compared to the first-generation compound. The authors showed that co-injection with 15 nmol of AlF-NOTA-2x(DV1c11sc12s) reduced uptake in liver and spleen by more than 10-fold while maintaining tumor uptake (SUV_mean_: 0.42 ± 0.16 vs. 0.47 ± 0.09). Despite reduced uptake in normal organs, tumor-to-organ ratios remain suboptimal for PET imaging compared to other classes of radiopharmaceuticals. Competition assays have shown no cross-reactivity of DV1/DV3 peptides with CCR5; however, selectivity against other GPCRs expressed in normal organs warrants further investigation.

## 5 Discussion

Most CXCR4 radiopharmaceuticals have been developed as PET imaging agents, whereas only a limited number have progressed towards therapeutic applications. CXCR4-targeted radiopharmaceuticals encompass a broad chemically landscape, including different scaffolds, linkers, chelators and radioisotopes. Optimizing each of these components is critical to achieving high sensitivity and specificity for PET imaging and achieving high tumor radiation dose delivery for effective RPT.

### 5.1 Challenges and opportunities in drug design

The availability of high-resolution receptor structures in their inactive, active and oligomeric states has opened new avenues for computer-assisted drug design. The integration of high-resolution structural information with large chemical libraries and AI approaches may accelerate discovery and optimization of next-generation CXCR4-targeting scaffolds (Lenselink et al., 2016; Mishra et al., 2016; Michino et al., 2025). Some key features of CXCR4 pharmacophores that have shown to be essential for receptor binding include basic amines, aromatic rings and hydrophobic moieties. The successful development of CXCR4 radiotheranostics requires balancing these features with appropriate charge distribution, polarity, and lipophilicity to maximize target engagement while promoting rapid clearance from normal tissues. Multimerization strategies have improved tumor uptake for selected radioligands, but their effects on receptor affinity, pharmacokinetics and non-specific uptake are highly scaffold-dependent. Linker charge and length optimization have been explored in only a few instances. For example, *in vitro* binding of [^68^Ga]Ga-Pentixafor was improved by incorporating a cationic linker, however, it resulted in increased renal uptake (Osl et al., 2020). For LY2510924-derived radiopharmaceuticals, *in vivo* data showed that anionic linkers significantly reduced renal uptake while not impairing binding (Kwon et al., 2022c). While neutral or anionic linkers are typically preferred to reduce renal uptake primarily through megalin-cubilin reabsorption mechanisms, this approach may not be suitable for all scaffolds. Although docking and SAR studies are helpful tools for rationalizing chemical modifications, advanced *in vitro* modeling and *in vivo* validation are necessary to ensure an optimal biodistribution. Binding assays have been instrumental in the development of CXCR4 radiotheranostics but the absence of standardized protocols limits direct comparison between reported compounds. Relaying on competitive inhibition assays may systematically underestimate allosteric binders or overestimate orthosteric binders depending on assay conditions. Furthermore, next-generation radiopharmaceuticals must be screened for receptor selectivity to prevent sequestration by secondary receptors.

### 5.2 Challenges and opportunities for clinical translation

The most extensive clinical experience has been achieved with radiolabeled peptides derived from the FC131 backbone (Pentixafor/Pentixather). However, a major limitation for clinical adoption of CXCR4 RPT is the associated bone marrow toxicity. Although pre-therapeutic human dosimetry results for [^177^Lu]Lu-Pentixather have shown heterogeneous results, significantly higher estimated absorbed doses in kidneys (0.38–3.5 mGy/MBq), spleen (0.34–2.26 mGy/MBq) and bone marrow (0.14–2.3 mGy/MBq) are observed compared to mice-extrapolated data (Schottelius et al., 2017; Hänscheid et al., 2021). This discrepancy highlights a major limitation of conventional murine models, where many CXCR4-targeted compounds exhibit preferential binding to human CXCR4 compared with murine CXCR4, resulting in underestimated uptake in human CXCR4-expressing tissues, particularly the hematopoietic compartment. Additional factors, including species-specific differences in CXCR4 expression patterns, receptor density, and transporter-mediated uptake mechanisms, may further contribute to discrepancies between preclinical and clinical observations. Standardized methods for dosimetry assessment and human extrapolation as well as more physiologically relevant models such as humanized or bone marrow engrafted mice are needed to identify therapeutic doses, radioprotective and bone marrow-sparing strategies.

A current strategy to mitigate bone marrow toxicity of CXCR4 RPT is using relatively short-lived radioisotopes in combination with HSC transplantation. The shorter half-life of ^90^Y compared to ^177^Lu allows for an earlier transplant after CXCR4 RPT which reduces the risk of severe infections and other complications (Buck et al., 2023). The half-life and energy emission properties of ^67^Cu and ^47^Sc make them promising alternatives within this approach. Also, emerging isotopes such as ^149/161^Tb, ^212^Pb, ^225^Ac and ^211^At may minimize radiation damage to the surrounding tissue due to the emission of short-range particles with a high linear energy transfer. However, their benefit and therapeutic index relative to ^90^Y and ^177^Lu in the context of CXCR4 RPT remains to be established. Strategies to reduce uptake in lymphoid organs have not been widely explored. Early studies suggest that a combinatorial therapy regime of CXCR4 RPT with non-radioactive CXCR4 targeting drugs may be a feasible approach to reduce the radiation dose deposited in lymphoid organs by producing a “blocking” effect in normal organs while maintaining tumor uptake (Spahn et al., 2024). Alternative combinatorial therapies with tumor-targeted radiosensitizers, DNA damage response inhibitors or immunomodulatory drugs, which may increase the therapeutic index of CXCR4 radioligands, have not yet been investigated. The emergence of alternative scaffolds, particularly LY2510924 and highly optimized chemokine-derived peptides, represent an important step toward next-generation CXCR4 radiotheranostics with improved chemical flexibility, favorable tumor-to-organ ratios, and robust isotope complexation properties. Future progress will depend on the continued development of next-generation scaffolds, well-motivated radioisotope selection and the availability of clinically relevant models that more accurately predict human pharmacokinetics and dosimetry. These advances will be key to improving tumor-to-organ ratios and achieving the therapeutic window required for successful CXCR4-targeted RPT.
