# Supplementary material for: Optimization of next-generation CXCR4 ligands for radiotheranostic applications
Source: Front Pharmacol. 2026 Sep 4;17:1923347. doi: 10.3389/fphar.2026.1923347 (PMC13587149; doi:10.3389/fphar.2026.1923347)
Supplement: Supplementary file 1 [file Supplementaryfile1.docx]

Supplementary Material

# 1 Supplementary Data

# Search query in the PubMed database for generation of Figure 1.

CXCR4 in drug development:

((("CXCR4"[tiab] OR "C-X-C motif chemokine receptor 4"[tiab]) AND (agonist* OR antagonist* OR inhibitor* OR ligand*) NOT (mechanism*[tiab] OR pathway*[tiab] OR gene*[tiab] OR mRNA*[tiab] OR "Signal Transduction"[Mesh] OR "Drug Resistance"[Mesh]))) NOT (Review[Publication Type])

CXCR4 radiopharmaceuticals:

((("CXCR4"[tiab] OR "C-X-C motif chemokine receptor 4"[tiab]) AND (agonist* OR antagonist* OR inhibitor* OR ligand*) NOT (mechanism*[tiab] OR pathway*[tiab] OR gene*[tiab] OR mRNA*[tiab] OR "Signal Transduction"[Mesh] OR "Drug Resistance"[Mesh])) **AND** (("**radioligand**") OR ("**radiopharmaceutical**") OR ("**radiotherapy**") OR ("**PET imaging**"))) NOT (Review[Publication Type])

# 2 Supplementary Figure

**Supplementary Figure 1.** Examples of the crystal structure of CXCR4:ligand complexes. A) CXCR4:CVX15 complex (PDB ID: 3OE0). B) CXCR4:IT1t complex (PDB ID: 3ODU). C) CXCR4:AMD3100 complex (PDB ID: 8ZPN) Polar interactions (dashed black lines), residues in the binding pocket (blue), CVX15 (purple), It1t (green), AMD3100 (yellow), water molecules (red sphere) and stabilizers (dark grey) are depicted. Structures were generated using PyMOL Molecular Graphics System, Version 3.2.0a Schrödinger, LLC.
